# Supplementary material for: Evaluation of microbiome and physico-chemical profiles of fresh fruits of Musa paradisiaca, Citrus sinensis and Carica papaya at different ripening stages: Implication to quality and safety management
Source: PLoS One. 2024 Jan 30;19(1):e0297574. doi: 10.1371/journal.pone.0297574 (PMC10826968; doi:10.1371/journal.pone.0297574)
Supplement: S1 File — (RTF) [file pone.0297574.s001.rtf]

Table 1. Mean microbial counts (Log CFU/mL) from unripe fruit samples 
No.	Sample Code	Source Sample	Mean microbial counts (Log CFU/mL)	
			TAMB	TSFB	LAB	Enterobacteriacaea	Staphylococcus 	Yeasts and Molds	
1.		BK1	Banana	6.30	4.02	4.10	3.2	3.60	4.01	
2.		BK2	Banana	7.27	4.90	4.80	0	3.60	4.2	
3.		BK3	Banana	7.02	4.80	4.00	3.2	0.00	4.1	
4.		BK4	Banana	6.69	5.10	5.08	0	0.00	3.34	
5.		BM1	Banana	7.30	4.74	4.80	3.1	2.70	3.36	
6.		BM2	Banana	7.30	4.77	4.10	0	3.00	3.31	
7.		BM3	Banana	7.10	4.68	5.20	3.7	2.90	4.2	
8.		BM4	Banana	6.30	4.02	5.30	3.4	3.10	4.04	
9.		BS1	Banana	6.40	5.01	6.20	3.6	3.00	4.3	
10.		BS2	Banana	6.29	5.25	5.04	3.7	0.00	4.2	
11.		BS3	Banana	6.40	4.80	3.60	3.9	3.00	4.2	
12.		BS4	Orange 	6.64	5.20	5.01	0	0.00	3.18	
13.		OK1	Orange 	6.20	4.04	4.70	3.3	0.00	4	
14.		OK2	Orange 	7.10	3.82	4.85	3.3	3.80	3.4	
15.		OK3	Orange 	7.10	4.89	5.25	0	0.00	4.04	
16.		OK4	Orange 	6.03	4.10	5.23	3.1	3.21	3.17	
17.		OM1	Orange 	6.30	3.60	5.20	0	3.18	4.2	
18.		OM2	Orange 	7.10	3.90	4.96	3	3.16	4	
19.		OM3	Orange 	7.20	4.92	5.01	3.3	3.26	3.3	
20.		OM4	Orange 	6.30	4.04	4.57	3.1	3.10	3.1	
21.		OS1	Orange 	7.20	5.00	5.00	0	0.00	3.9	
22.		OS2	Orange 	7.20	3.90	5.10	0	3.12	4.3	
23.		OS3	Orange 	7.10	4.00	4.50	3.3	0.00	4.3	
24.		OS4	Orange 	6.3	4.91	5.01	0	3.26	3.12	
25.		PK1	Papaya 	7.10	4.74	4.90	3.3	0.00	3.9	
26.		PK2	Papaya 	6.30	4.07	4.90	3.4	3.15	4.2	
27.		PK3	Papaya 	7.20	4.20	5.70	3.3	0.00	3.98	
28.		PK4	Papaya 	7.3	5.50	5.15	3.14	3.11	4.12	
29.		PM1	Papaya 	6.30	5.00	4.70	0	3.19	4.3	
30.		PM2	Papaya 	7.20	4.92	5.03	3.1	2.90	4.2	
31.		PM3	Papaya 	7.20	4.90	5.15	3.4	0.00	4.2	
32.		PM4	Papaya 	6.20	4.20	5.10	3.3	3.30	3.5	
33.		PS1	Papaya 	7.20	4.00	5.24	3.2	2.80	4.2	
34.		PS2	Papaya 	6.30	4.20	4.90	3.4	3.14	4.27	
35.		PS3	Papaya 	6.20	5.00	4.70	3.4	3.17	4.25	
36.		PS4	Papaya 	7.02	4.80	5.24	0	0.00	3.9	

Table 2. Mean microbial counts (Log CFU/mL) from moderately ripened fruit samples
No.	Sample Code	Source Sample	Mean microbial counts (Log CFU/mL)	
			TAMB	TSFB	LAB	Enterobacteriacaea	Staphylococcus	Yeasts and Molds	
1.		2BK1	Banana	7.80	6.00	5.12	2.5	3.80	5.00	
2.		2BK2	Banana	7.90	5.90	5.26	0	3.50	4.20	
3.		2BK3	Banana	8.00	6.10	6.01	4.2	3.60	4.10	
4.		2BK4	Banana	7.20	6.00	5.07	3.00	3.00	3.55	
5.		2BM1	Banana	7.30	6.70	4.30	4	2.90	4.30	
6.		2BM2	Banana	7.00	6.10	6.08	0	3.00	4.30	
7.		2BM3	Banana	8.00	6.70	5.27	4.1	2.83	4.20	
8.		2BM4	Banana	7.30	5.90	6.04	3.1	2.80	4.90	
9.		2BS1	Banana	7.60	5.20	6.15	4.6	3.62	4.30	
10.		2BS2	Banana	8.30	6.10	5.93	2.5	2.90	4.20	
11.		2BS3	Banana	7.70	6.00	6.10	4	3.66	4.20	
12.		2BS4	Orange 	7.10	6.10	6.00	3.1	3.40	3.90	
13.		2OK1	Orange 	7.00	5.20	5.00	3.3	0.00	4.40	
14.		2OK2	Orange 	6.30	5.30	4.30	3.3	3.62	3.40	
15.		2OK3	Orange 	7.30	5.30	5.30	3.3	0.00	4.00	
16.		2OK4	Orange 	6.70	5.90	5.90	2.9	3.12	3.29	
17.		2OM1	Orange 	6.40	5.30	5.10	0	3.70	4.20	
18.		2OM2	Orange 	6.30	5.10	5.12	3	2.80	4.00	
19.		2OM3	Orange 	7.20	5.20	5.15	3.3	3.71	3.30	
20.		2OM4	Orange 	7.30	6.10	6.10	3.1	3.29	4.00	
21.		2OS1	Orange 	6.30	5.20	5.09	0	0.00	4.00	
22.		2OS2	Orange 	6.40	5.30	5.06	0	2.78	4.30	
23.		2OS3	Orange 	7.30	5.90	5.91	0	3.55	4.30	
24.		2OS4	Orange 	7.20	5.10	5.95	2.88	0.00	3.71	
25.		2PK1	Papaya 	7.30	5.20	6.03	3.4	3.83	4.00	
26.		2PK2	Papaya 	7.00	5.20	5.30	3.4	2.94	4.20	
27.		2PK3	Papaya 	7.40	6.20	6.21	3.3	3.81	4.00	
28.		2PK4	Papaya 	6.80	5.10	5.24	2.93	3.71	3.80	
29.		2PM1	Papaya 	7.30	5.30	5.97	3.5	2.90	4.30	
30.		2PM2	Papaya 	7.30	5.30	5.66	3.1	3.55	4.20	
31.		2PM3	Papaya 	7.90	5.30	5.25	3.5	2.91	4.20	
32.		2PM4	Papaya 	7.20	6.10	6.01	4	3.60	4.10	
33.		2PS1	Papaya 	7.70	6.00	6.15	3.2	3.00	4.20	
34.		2PS2	Papaya 	7.30	5.20	6.06	3.4	2.94	4.90	
35.		2PS3	Papaya 	7.00	5.30	5.28	3.4	3.03	4.90	
36.		2PS4	Papaya 	6.80	6.00	5.96	2.96	0.00	3.83	

Table 3. Mean microbial counts (Log CFU/mL) from overripe fruit samples
No.	Sample Code	Source Sample	Mean microbial counts (Log CFU/mL)	
			TAMB	TSFB	LAB	Enterobacteriacaea	Staphylococcus 	Yeasts and Molds	
1.		3BK1	Banana	8.23	6.96	5.91	3.02	3.16	5.04	
2.		3BK2	Banana	8.00	7.03	6.05	0.00	3.01	5.12	
3.		3BK3	Banana	8.27	6.80	6.09	3.23	3.64	4.24	
4.		3BK4	Banana	7.90	6.30	6.04	0.00	3.28	5.04	
5.		3BM1	Banana	8.05	7.12	5.27	3.16	3.23	5.11	
6.		3BM2	Banana	7.25	7.07	6.28	0.00	3.73	4.96	
7.		3BM3	Banana	8.29	7.05	6.25	3.03	3.26	4.95	
8.		3BM4	Banana	8.06	7.10	6.03	3.18	3.91	4.94	
9.		3BS1	Banana	7.30	6.82	6.30	0.00	3.29	5.12	
10.		3BS2	Banana	7.97	6.93	6.94	3.25	3.30	5.02	
11.		3BS3	Banana	7.93	6.26	5.79	3.13	3.66	4.94	
12.		3BS4	Orange 	8.21	7.00	6.30	3.00	3.02	5.10	
13.		3OK1	Orange 	7.89	6.02	5.93	0.00	3.49	4.09	
14.		3OK2	Orange 	7.21	5.88	5.19	3.01	3.11	4.03	
15.		3OK3	Orange 	7.04	6.04	5.84	3.04	3.13	3.10	
16.		3OK4	Orange 	7.07	6.29	5.28	0.00	3.06	3.72	
17.		3OM1	Orange 	7.24	6.22	6.03	0.00	3.01	4.08	
18.		3OM2	Orange 	7.07	6.01	5.26	3.08	3.67	4.99	
19.		3OM3	Orange 	7.32	6.03	5.29	0.00	3.30	4.19	
20.		3OM4	Orange 	7.93	6.05	5.99	3.03	3.96	4.02	
21.		3OS1	Orange 	8.02	6.07	6.26	3.12	3.58	3.86	
22.		3OS2	Orange 	7.98	6.09	5.97	0.00	3.92	4.26	
23.		3OS3	Orange 	8.06	6.12	6.02	3.03	3.81	3.97	
24.		3OS4	Orange 	7.23	6.03	5.05	3.01	3.85	4.08	
25.		3PK1	Papaya 	7.76	6.06	5.83	0.00	3.78	4.06	
26.		3PK2	Papaya 	8.03	6.09	6.24	3.21	3.90	4.98	
27.		3PK3	Papaya 	7.26	6.10	5.90	0.00	3.20	5.05	
28.		3PK4	Papaya 	8.04	6.88	6.29	3.12	3.94	4.92	
29.		3PM1	Papaya 	7.89	6.01	6.21	3.24	3.69	5.06	
30.		3PM2	Papaya 	8.16	6.12	5.99	3.17	3.98	4.95	
31.		3PM3	Papaya 	7.09	6.15	6.16	0.00	3.71	5.03	
32.		3PM4	Papaya 	8.07	6.13	6.13	3.28	3.90	4.08	
33.		3PS1	Papaya 	7.92	6.98	6.26	3.21	3.87	4.98	
34.		3PS2	Papaya 	7.90	6.07	6.28	0.00	3.08	5.09	
35.		3PS3	Papaya 	7.91	6.23	6.25	3.14	4.03	5.05	
36.		3PS4	Papaya 	7.77	6.03	6.08	0.00	3.71	4.21	
