# Supplementary material for: Evaluation of microbiome and physico-chemical profiles of fresh fruits of Musa paradisiaca, Citrus sinensis and Carica papaya at different ripening stages: Implication to quality and safety management
Source: PLoS One. 2024 Jan 30;19(1):e0297574. doi: 10.1371/journal.pone.0297574 (PMC10826968; doi:10.1371/journal.pone.0297574)
Supplement: S2 File — (RTF) [file pone.0297574.s002.rtf]

Table 4. Morphological and Biochemical Characterization of Bacterial isolates from unripe fruit samples
No	Isolate  Code	Colony Morphology on NA	Cell Shape	Gram Reaction	Motility	Endospore	Indole	H2S	TSIA	Catalase	Citrate	Coagulase 	Oxidase 	Urease	Presumptive  Species	
		Color 	Shape	Texture	Elev. 	Size 														
1.		BK1B2	White 	Circular 	Rough  	Raised 	Large 	Rod	+	+	+	-	-	+	+	+	- 	+	+	Bacillus Spp	
2.		BK2B3	Creamy 	Circular 	Smooth 	Flat	Large 	Rod 	+	+	+	-	-	+	+	+	- 	+	-	Bacillus Spp	
3.		BK4B4	Gray 	Circular 	Smooth 	Flat	Medium 	Rod	+	+	+	-	-	+	+	+	- 	+	+	Bacillus Spp	
4.		BM1B2	Creamy 	Circular 	Smooth 	Flat	Medium 	Rod	+	+	+	-	-	+	+	+	- 	+	-	Bacillus Spp	
5.		BM4B1	White 	Circular 	Rough  	Raised 	Large 	Rod	+	+	+	-	-	+	+	+	- 	+	+	Bacillus Spp	
6.		BS1B1	Creamy 	Circular 	Smooth 	Flat	Medium 	Rod	+	+	+	-	-	+	+	+	- 	+	-	Bacillus Spp	
7.		BS3B2	Creamy 	Circular 	Smooth 	Flat 	Medium 	Rod	+	+	+	-	-	+	+	+	- 	+	-	Bacillus Spp	
8.		OK2B3	White 	Circular 	Rough  	Raised	Large 	Rod	+	+	+	-	-	+	+	+	- 	+	+	Bacillus Spp	
9.		OK3B4	Creamy 	Circular 	Smooth 	Flat	Medium 	Rod	+	+	+	-	-	+	+	+	- 	+	-	Bacillus Spp	
10.		OM1B2	Creamy 	Circular 	Smooth 	Flat	Medium 	Rod	+	+	+	-	-	+	+	+	- 	+	-	Bacillus Spp	
11.		OM4B3	White 	Circular 	Rough  	Raised 	Large 	Rod	+	+	+	-	-	+	+	+	- 	+	+	Bacillus Spp	
12.		OS2B4	Creamy 	Circular 	Smooth 	Flat	Medium 	Rod	+	+	+	-	-	+	+	+	- 	+	-	Bacillus Spp	
13.		PK1B2	Creamy 	Circular 	Smooth 	Flat	Medium 	Rod	+	+	+	-	-	+	+	+	- 	+	-	Bacillus Spp	
14.		PK2B1	White 	Circular 	Rough  	Raised 	Large 	Rod	+	+	+	-	-	+	+	+	- 	+	+	Bacillus Spp	
15.		PK4B3	Creamy 	Circular 	Smooth 	Flat	Medium 	Rod	+	+	+	-	-	+	+	+	- 	+	-	Bacillus Spp	
16.		PM1B4	Gray 	Circular 	Smooth 	Flat	Medium 	Rod	+	+	+	-	-	+	+	+	- 	+	+	Bacillus Spp	
17.		PM2B1	Creamy 	Circular 	Smooth 	Flat	Medium 	Rod	+	+	+	-	-	+	+	+	- 	+	-	Bacillus Spp	
18.		PM4B2	Creamy 	Circular 	Smooth 	Flat	Medium 	Rod	+	+	+	-	-	+	+	+	- 	+	-	Bacillus Spp	
19.		PS2B3	Creamy 	Circular 	Smooth 	Flat	Medium 	Rod	+	+	+	-	-	+	+	+	- 	+	-	Bacillus Spp	
20.		PS4B3	Gray 	Circular 	Smooth 	Flat	Medium 	Rod	+	+	+	-	-	+	+	+	- 	+	+	Bacillus Spp	
21.		BK1B4	White	Circular 	Smooth 	Raised 	Small 	Coccus 	+	-	-	-	-	+	+	+	-	+	+	Staphylococcus Spp	
22.		BM2B1	White	Circular 	Smooth 	Raised 	Small 	Coccus 	+	-	-	-	-	+	+	+	+	-	-	Staphylococcus Spp	
23.		BS1B1	White	Circular 	Smooth 	Raised 	Small 	Coccus 	+	-	-	-	-	+	+	+	+	-	-	Staphylococcus Spp	
24.		BM2B4	White	Circular 	Smooth 	Raised 	Small 	Coccus 	+	-	-	-	-	+	+	+	-	+	+	Staphylococcus Spp	
25.		BM4B1	White	Circular 	Smooth 	Raised 	Small 	Coccus 	+	-	-	-	-	+	+	+	-	+	+	Staphylococcus Spp	
26.		OK2B2	White	Circular 	Smooth 	Raised 	Small 	Coccus 	+	-	-	-	-	+	+	+	+	-	-	Staphylococcus Spp	
27.		OM1B4	White	Circular 	Smooth 	Raised 	Small 	Coccus 	+	-	-	-	-	+	+	+	+	-	-	Staphylococcus Spp	
28.		OS4B3	White	Circular 	Smooth 	Raised 	Small 	Coccus 	+	-	-	-	-	+	+	+	-	+	+	Staphylococcus Spp	
29.		PK2B1	White	Circular 	Smooth 	Raised 	Small 	Coccus 	+	-	-	-	-	+	+	+	-	+	+	Staphylococcus Spp	
30.		PS1B2	White	Circular 	Smooth 	Raised 	Small 	Coccus 	+	-	-	-	-	+	+	+	+	-	-	Staphylococcus Spp	
31.		PS3B5	White	Circular 	Smooth 	Raised 	Small 	Coccus 	+	-	-	-	-	+	+	+	+	-	-	Staphylococcus Spp	
32.		PM1B4	White	Circular 	Smooth 	Raised 	Small 	Coccus 	+	-	-	-	-	+	+	+	+	-	-	Staphylococcus Spp	
33.		PM4B1	White	Circular 	Smooth 	Raised 	Small 	Coccus 	+	-	-	-	-	+	+	+	-	+	+	Staphylococcus Spp	
34.		PS2B2	White	Circular 	Smooth 	Raised 	Small 	Coccus 	+	-	-	-	-	+	+	+	-	+	+	Staphylococcus Spp	
35.		BK1B3	Gray  	Circular 	Smooth 	Raised 	Medium 	Rod	-	+	-	+	+	-	+	-	-	+	-	E. coli	
36.		BM2B2	Creamy 	Circular 	Smooth 	Raised 	Medium 	Rod	-	+	-	+	+	-	+	-	-	+	-	E. coli	
37.		BS2B3	Creamy 	Circular 	Smooth 	Raised 	Medium 	Rod	-	+	-	+	+	-	+	-	-	+	-	E. coli	
38.		OM1B2	Creamy 	Circular 	Smooth 	Raised 	Medium 	Rod	-	+	-	+	+	-	+	-	-	+	-	E. coli	
39.		OM2B5	Creamy 	Circular 	Smooth 	Raised 	Medium 	Rod	-	+	-	+	+	-	+	-	-	+	-	E. coli	
40.		PK1B3	Gray  	Circular 	Smooth 	Raised 	Medium 	Rod	-	+	-	+	+	-	+	-	-	+	-	E. coli	
41.		PM1B1	Gray  	Circular 	Smooth 	Raised 	Medium 	Rod	-	+	-	+	+	-	+	-	-	+	-	E. coli	
42.		PM4B3	Creamy 	Circular 	Smooth 	Raised 	Medium 	Rod	-	+	-	+	+	-	+	-	-	+	-	E. coli	
43.		PS2B1	Creamy 	Circular 	Smooth 	Raised 	Medium 	Rod	-	+	-	+	+	-	+	-	-	+	-	E. coli	
44.		OK1B1	White	Circular 	Smooth 	Raised 	Small 	Rod	-	+	-	-	+	-	+	+	-	+	-	Pseudomonas Spp	
45.		OM1B1	White	Circular 	Smooth 	Raised 	Small 	Rod	-	+	-	+	+	-	+	+	-	+	-	Pseudomonas Spp	
46.		OS4B2	White	Circular 	Smooth 	Raised 	Small 	Rod	-	+	-	-	+	-	+	+	-	+	+	Pseudomonas Spp	
47.		BK2B5	Creamy 	Circular 	Smooth 	Raised 	Medium 	Rod	-	+	-	-	+	+	+	+	-	+	-	Salmonella Spp	
48.		BM1B1	Creamy 	Circular 	Smooth 	Raised 	Medium 	Rod	-	+	-	-	+	+	+	+	-	+	-	Salmonella Spp	
49.		OM1B2	Creamy 	Circular 	Smooth 	Raised 	Medium 	Rod	-	+	-	-	+	+	+	+	-	+	-	Salmonella Spp	
50.		PK2B2	Creamy 	Circular 	Smooth 	Raised 	Medium 	Rod	-	+	-	-	+	+	+	+	-	+	-	Salmonella Spp	
51.		PM2B1	Creamy 	Circular 	Smooth 	Raised 	Medium 	Rod	-	+	-	-	+	+	+	+	-	+	-	Salmonella Spp	
52.		PS1B4	Creamy 	Circular 	Smooth 	Raised 	Medium 	Rod	-	+	-	-	+	+	+	+	-	+	-	Salmonella Spp	
53.		BK4B1	Creamy 	Circular 	Smooth 	Raised 	Medium 	Rod	-	-	-	-	-	+	+	-	-	+	-	Shigella Spp	
54.		BM2B1	Creamy 	Circular 	Smooth 	Raised 	Medium 	Rod	-	-	-	-	-	+	+	-	-	+	-	Shigella Spp	
55.		PM2B2	Creamy 	Circular 	Smooth 	Raised 	Medium 	Rod	-	-	-	-	-	+	+	-	-	+	-	Shigella Spp	
56.		BK3B1	Red 	Circular 	Smooth 	Raised 	Medium 	Rod	-	+	-	-	-	+	+	-	-	-	-	Serratia Spp	
Table 5. Morphological and biochemical characterization of yeasts isolates from unripe fruit samples
No	
Isolate 	Colony morphology on SDA	Colony color on BiGGY Agar	GT Test	Presumptive  Species	
		Color	Margin	Texture	Size 	Elevation				
1.		BK1Y2	Creamy 	Circular	Smooth	Small	Flat	Dark brown/yellow halo	-	Candida Spp.	
2.		BK2Y2	Creamy 	Circular	Smooth	Medium	Flat	Brown/black center	-	Candida Spp.	
3.		BK3Y3	Creamy 	Circular	Smooth	Medium	Flat	Dark brown/yellow halo	-	Candida Spp.	
4.		BK4Y1	Creamy 	Circular	Smooth	Small	Flat	Dark brown/yellow halo	+	Candida Spp.	
5.		BM1Y2	Creamy 	Circular	Smooth	Medium	Flat	Brown/black center	-	Candida Spp.	
6.		BM2Y3	Creamy 	Circular	Smooth	Small	Flat	Brown/black center	-	Candida Spp.	
7.		BM3Y3	Creamy 	Circular	Smooth	Small	Flat	Dark brown/yellow halo	-	Candida Spp.	
8.		BM4Y4	Creamy 	Circular	Smooth	Large	Flat	Brown/black center	-	Candida Spp.	
9.		BS1Y2	Creamy 	Circular	Smooth	Small	Convex	Light brown	+	Candida Spp.	
10.		BS2Y1	Creamy 	Circular	Smooth	Small	Flat	Dark brown/yellow halo	-	Candida Spp.	
11.		BS3Y1	Creamy 	Circular	Smooth	Large	Flat	Light brown	+	Candida Spp.	
12.		BS3Y3	Creamy 	Circular	Smooth	Large	Convex	Brown/black center	-	Candida Spp.	
13.		BS4Y4	Creamy 	Circular	Smooth	Medium	Flat	Dark brown/yellow halo	-	Candida Spp.	
14.		OK1Y2	Creamy 	Circular	Smooth	Large	Convex 	Light brown	+	Candida Spp.	
15.		OK1Y5	Creamy 	Circular	Smooth	Small	Fla	Dark brown/yellow halo	-	Candida Spp.	
16.		OK2Y1	Creamy 	Circular	Smooth	Large	Convex 	Dark brown/yellow halo	-	Candida Spp.	
17.		OK2Y4	Creamy 	Circular	Smooth	Small	Flat 	Dark brown/yellow halo	+	Candida Spp.	
18.		OK3Y2	Creamy 	Circular	Smooth	Large	Convex 	Brown/black center	-	Candida Spp.	
19.		OM2Y1	Creamy 	Circular	Smooth	Small	Flat 	Dark brown/yellow halo	-	Candida Spp.	
20.		OM3Y2	Creamy 	Circular	Smooth	Medium	Flat 	Dark brown/yellow halo	-	Candida Spp.	
21.		OM3Y5	Creamy 	Circular	Smooth	Small	Flat 	Brown/black center	-	Candida Spp.	
22.		OM4Y1	Creamy 	Circular	Smooth	Medium	Flat 	Brown/black center	-	Candida Spp.	
23.		OS1Y3	Creamy 	Circular	Smooth	Small	Flat 	Brown/black center	-	Candida Spp.	
24.		OS2Y4	Creamy 	Circular	Smooth	Small	Convex 	Dark brown/yellow halo	-	Candida Spp.	
25.		OS3Y5	Creamy 	Circular	Smooth	Large	Flat	Light brown	-	Candida Spp.	
26.		PK1Y1	Creamy 	Circular	Smooth	Small	Flat	Brown/black center	+	Candida Spp.	
27.		PK1Y2	Creamy 	Circular	Smooth	Small	Convex 	Dark brown/yellow halo	-	Candida Spp.	
28.		PK2Y3	Creamy 	Circular	Smooth	Large	Convex 	Dark brown	-	Candida Spp.	
29.		PK4Y2	Creamy 	Circular	Smooth	Small	Flat 	Dark brown/yellow halo	-	Candida Spp.	
30.		PM1Y1	Creamy 	Circular	Smooth	Small	Flat 	Brown/black center	+	Candida Spp.	
31.		PM1Y2	Creamy 	Circular	Smooth	Medium	Flat 	Brown/black center	-	Candida Spp.	
32.		PM2Y1	Creamy 	Circular	Smooth	Small	Flat 	Brown/black center	+	Candida Spp.	
33.		PM3Y2	Creamy 	Circular	Smooth	Large	Flat	Dark brown/yellow halo	-	Candida Spp.	
34.		PM4Y1	Creamy 	Circular	Smooth	Large	Flat 	Dark brown	+	Candida Spp.	
35.		PS1Y1	Creamy 	Circular	Smooth	Large	Convex 	Dark brown/yellow halo	-	Candida Spp.	
36.		PS2Y1	Creamy 	Circular	Smooth	Small	Convex 	Dark brown/yellow halo	-	Candida Spp.	
37.		PS3Y2	Creamy 	Circular	Smooth	Large	Flat	Dark brown	-	Candida Spp.	
38.		PS4Y2	Creamy 	Circular	Smooth	Small	Convex 	Dark brown	+	Candida Spp.	
39.		BK1Y4	White 	Circular	Smooth	Small	Convex 	White shiny	-	Saccharomyces  Spp.	
40.		BK2Y1	White 	Circular	Smooth	Small	Convex 	Creamy	-	Saccharomyces  Spp.	
41.		BK3Y3	White 	Circular	Smooth	Small	Convex 	Light brown	-	Saccharomyces  Spp.	
42.		BM1Y1	White 	Circular	Smooth	Small	Convex 	Creamy	-	Saccharomyces  Spp.	
43.		BM3Y2	White 	Circular	Smooth	Small	Convex 	Light brown	-	Saccharomyces  Spp.	
44.		BM4Y4	White 	Circular	Smooth	Medium	Convex 	Creamy	-	Saccharomyces  Spp.	
45.		BS1Y2	White 	Circular	Smooth	Small	Convex 	Creamy	-	Saccharomyces  Spp.	
46.		BS3Y1	White 	Circular	Smooth	Small	Convex 	White shiny	-	Saccharomyces  Spp.	
47.		OK1Y4	White 	Circular	Smooth	Medium	Convex 	White shiny	-	Saccharomyces  Spp.	
48.		OK4Y5	White 	Circular	Smooth	Small	Convex 	White shiny	-	Saccharomyces  Spp.	
49.		OM2Y2	White 	Circular	Smooth	Small	Convex 	Creamy	-	Saccharomyces  Spp.	
50.		OM2Y3	White 	Circular	Smooth	Small	Convex 	Creamy	-	Saccharomyces  Spp.	
51.		OS4Y2	White 	Circular	Smooth	Small	Convex 	Light brown	-	Saccharomyces  Spp.	
52.		PK1Y4	White 	Circular	Smooth	Small	Convex 	Light brown	-	Saccharomyces  Spp.	
53.		PK2Y2	White 	Circular	Smooth	Small	Convex 	Light brown	-	Saccharomyces  Spp.	
54.		PM1Y5	White 	Circular	Smooth	Small	Convex 	White shiny	-	Saccharomyces  Spp.	
55.		PM2Y4	White 	Circular	Smooth	Medium	Convex 	White shiny	-	Saccharomyces  Spp.	
56.		PM3Y3	White 	Circular	Smooth	Small	Convex 	Light brown	-	Saccharomyces  Spp.	
57.		PS2Y4	White 	Circular	Smooth	Small	Convex 	Creamy	-	Saccharomyces  Spp.	
58.		PS4Y3	White 	Circular	Smooth	Small	Convex 	Creamy	-	Saccharomyces  Spp.	

Table 6. Morphological Characterization of mold isolates from unripe fruit samples grown on PDA
No	Isolate Code	Front colony  color	Reverse side  color	Spore Color 	Growth Pattern	Presumptive  Species	
1.		BK1M2	Dark gray	Deep black	Gray	Dom/Fast	Alternaria Spp.	
2.		BM2M1	Dark gray	Deep black	Gray	Dom/Fast	Alternaria Spp.	
3.		BS4M2	Dark gray	Deep black	Gray	Dom/Fast	Alternaria Spp.	
4.		OM1M4	Dark gray	Light brown	White 	Dom/Fast	Alternaria Spp.	
5.		OM1M7	Dark gray	Deep black	Gray	Dom/Fast	Alternaria Spp.	
6.		PK1M3	Dark gray	Deep black	Gray	Dom/Fast	Alternaria Spp.	
7.		PK2M1	Dark gray	Deep black	Gray	Dom/Fast	Alternaria Spp.	
8.		PM1M3	Dark gray	Deep black	Gray	Dom/Fast	Alternaria Spp.	
9.		PM2M3	Dark gray	Deep black	Gray	Dom/Fast	Alternaria Spp.	
10.		PS2M1	Dark gray	Deep black	Gray	Dom/Fast	Alternaria Spp.	
11.		PS4M1	Dark gray	Deep black	Gray	Dom/Fast	Alternaria Spp.	
12.		BK1M1	Sand-brown	Dirty-Brown 	Brown	Dom/flat	Aspergillus Spp.	
13.		BK2M2	Black	Dirty grey	Gray	Dom/flat	Aspergillus Spp.	
14.		BM2M3	Green	Dark Green	Gray	Dom/flat	Aspergillus Spp.	
15.		BM3M1	Black	Dirty grey	Gray	Dom/flat 	Aspergillus Spp.	
16.		BS2M1	Sand-brown	Dirty-Brown 	Brown	Dom/flat	Aspergillus Spp.	
17.		BS4M3	Sand-brown	Dirty-Brown 	Brown	Dom/flat	Aspergillus Spp.	
18.		OK3M1	Gray Green  	Creamy	Gray	Dom/flat	Aspergillus Spp.	
19.		OM1M1	Green	Dark Green	Gray	Dom/flat	Aspergillus Spp.	
20.		OS1M3	Gray Green	Creamy	Gray	Dom/flat	Aspergillus Spp.	
21.		OS2M3	Gray Green	Creamy	Gray	Dom/flat	Aspergillus Spp.	
22.		PM1M1	Sand-brown	Dirty-Brown 	Brown	Dom/Fast	Aspergillus Spp.	
23.		PM2M1	Sand-brown	Dirty-Brown 	Brown	Dom/Fast	Aspergillus Spp.	
24.		PM3M2	Sand-brown	Dirty-Brown 	Brown	Dom/flat	Aspergillus Spp.	
25.		PS1M1	Gray Green	Creamy	Gray	Dom/flat	Aspergillus Spp.	
26.		PS2M1	Black 	Dirty grey	Gray	Dom/flat 	Aspergillus Spp.	
27.		PS4M1	Green	Dark Green	Gray	Dom/flat	Aspergillus Spp.	
28.		BK2M6	White	Light brown	Gray 	Midium	Botrytis Spp.	
29.		BM2M4	White	Light brown	Gray	Midium	Botrytis Spp.	
30.		OK1M2	White/grey sporan	Light brown	White 	Midium	Botrytis Spp.	
31.		OS2M1	White/ Gray sporang 	Light brown	White 	Dom/flat	Botrytis Spp.	
32.		PK2M1	Yellow/white margin	Light brown	Yellow	Midium	Botrytis Spp.	
33.		PM1M2	White/grey sporan	Light brown	White 	Midium	Botrytis Spp.	
34.		PS2M3	White/grey sporan	Light brown	Gray 	Midium	Botrytis Spp.	
35.		BK2M2	Pinkish yellow	Dirty borwn	Yellow	Slow	Fusarium Spp.	
36.		BM1M1	Pinkish yellow	Dirty borwn	Yellow	Midium	Fusarium Spp.	
37.		BM2M3	Pinkish yellow	Dirty borwn	Yellow	Slow 	Fusarium Spp.	
38.		PS1M4	Pink/Cloudy	Pinkish brown	Cloudy	Midium	Fusarium Spp.	
39.		BK1M1	White	White 	White 	Dom/Fast	Mucor Spp.	
40.		BM1M4	White	White 	White 	Dom/Fast	Mucor Spp.	
41.		BS1M3	White	White 	White 	Dom/Fast	Mucor Spp.	
42.		BS2M7	Yellow/cloudy	Creamy brown	Cloudy	Slow	Mucor Spp.	
43.		PM2M2	White	White 	White 	Dom/Fast	Mucor Spp.	
44.		BK1M3	Grayish green	Creamy   	Gray	Dom/Fast	Penicillium Spp.	
45.		BM4M1	Grayish green	Creamy 	Gray	Dom/Fast	Penicillium Spp.	
46.		OK2M1	Grayish green	Creamy	Gray	Dom/Fast	Penicillium Spp.	
47.		OM2M2	Grayish green	Creamy	Gray	Dom/Fast	Penicillium Spp.	
48.		PM2M4	Grayish green	Creamy	Gray	Dom/Fast	Penicillium Spp.	
49.		BK2M6	White/black spots 	Light brown	Black 	Dom/Fast	Rhzopus Spp.	
50.		BM2M1	White/black spots	Light brown	Black 	Dom/Fast	Rhzopus Spp.	
51.		OM1M3	White/black spots	Light brown	Black 	Dom/Fast	Rhzopus Spp.	

Table 7.  Morphological and biochemical characterization of bacterial isolates from moderately ripened fruit samples
No	Isolate/ Code	Colony Morphology on NA	
Cell Shape	Gram Reaction	Motility	Endospore 	Indole	H2S	TSIA	Catalase	Citrate	Coagulase 	Oxidase 	Urease	
Presumptive  Species	
		Color 	Shape	Texture	Elev. 	Size 														
1.		2BK1B1	Creamy 	Circular 	Smooth 	Flat	Medium 	Rod	+	+	+	-	-	+	+	+	- 	+	-	Bacillus Spp	
2.		2BK3B1	White  	Circular 	Rough  	Raised 	Large 	Rod	+	+	+	-	-	+	+	+	- 	+	+	Bacillus Spp	
3.		2BS2B1	Gray 	Circular 	Smooth 	Flat	Medium 	Rod	+	+	+	-	-	+	+	+	- 	+	+	Bacillus Spp	
4.		2BK4B4	Creamy 	Circular 	Smooth 	Flat	Medium 	Rod	+	+	+	-	-	+	+	+	- 	+	-	Bacillus Spp	
5.		2BM1B1	White 	Circular 	Rough  	Raised 	Large 	Rod	+	+	+	-	-	+	+	+	- 	+	+	Bacillus Spp	
6.		2BM4B2	Creamy 	Circular 	Smooth 	Flat	Medium 	Rod	+	+	+	-	-	+	+	+	- 	+	-	Bacillus Spp	
7.		2BM2B4	Creamy 	Circular 	Smooth 	Flat 	Medium 	Rod	+	+	+	-	-	+	+	+	- 	+	-	Bacillus Spp	
8.		2BM3B5	White 	Circular 	Rough  	Raised	Large 	Rod	+	+	+	-	-	+	+	+	- 	+	+	Bacillus Spp	
9.		2BS2B3	Creamy 	Circular 	Smooth 	Flat	Medium 	Rod	+	+	+	-	-	+	+	+	- 	+	-	Bacillus Spp	
10.		2BS3B3	White  	Circular 	Rough  	Raised 	Large 	Rod	+	+	+	-	-	+	+	+	- 	+	+	Bacillus Spp	
11.		2BS4B1	Creamy 	Circular 	Smooth 	Flat	Medium 	Rod	+	+	+	-	-	+	+	+	- 	+	-	Bacillus Spp	
12.		2OK1B3	Creamy 	Circular 	Smooth 	Flat	Medium 	Rod	+	+	+	-	-	+	+	+	- 	+	-	Bacillus Spp	
13.		2OK2B3	Gray 	Circular 	Smooth 	Flat	Large 	Rod	+	+	+	-	-	+	+	+	- 	+	+	Bacillus Spp	
14.		2OK3B2	Creamy 	Circular 	Smooth 	Flat	Large 	Rod	+	+	+	-	-	+	+	+	- 	+	-	Bacillus Spp	
15.		2OM2B1	Creamy 	Circular 	Smooth 	Flat	Medium 	Rod	+	+	+	-	-	+	+	+	- 	+	-	Bacillus Spp	
16.		2OM3B1	Gray 	Circular 	Smooth 	Flat	Medium 	Rod	+	+	+	-	-	+	+	+	- 	+	+	Bacillus Spp	
17.		2OM4B1	Creamy 	Circular 	Smooth 	Flat	Medium 	Rod	+	+	+	-	-	+	+	+	- 	+	-	Bacillus Spp	
18.		2OS1B3	Gray 	Circular 	Smooth 	Flat	Medium 	Rod	+	+	+	-	-	+	+	+	- 	+	+	Bacillus Spp	
19.		2OS4B1	Creamy 	Circular 	Smooth 	Flat	Medium 	Rod	+	+	+	-	-	+	+	+	- 	+	-	Bacillus Spp	
20.		2PK1B4	White  	Circular 	Rough  	Raised 	Large 	Rod	+	+	+	-	-	+	+	+	- 	+	+	Bacillus Spp	
21.		2PK2B3	Gray 	Circular 	Smooth 	Flat	Medium 	Rod	+	+	+	-	-	+	+	+	- 	+	+	Bacillus Spp	
22.		2PK3B1	Creamy 	Circular 	Smooth 	Flat	Large 	Rod	+	+	+	-	-	+	+	+	- 	+	-	Bacillus Spp	
23.		2PK4B3	Creamy 	Circular 	Smooth 	Flat	Large 	Rod	+	+	+	-	-	+	+	+	- 	+	-	Bacillus Spp	
24.		2PM1B4	White  	Circular 	Rough  	Raised 	Large 	Rod	+	+	+	-	-	+	+	+	- 	+	+	Bacillus Spp	
25.		2PM2B3	Gray 	Circular 	Smooth 	Raised 	Medium 	Rod	+	+	+	-	-	+	+	+	- 	+	+	Bacillus Spp	
26.		2PM3B3	Gray 	Circular 	Smooth 	Raised 	Medium 	Rod	+	+	+	-	-	+	+	+	- 	+	+	Bacillus spp	
27.		2PM4B2	White  	Circular 	Rough  	Raised 	Large 	Rod	+	+	+	-	-	+	+	+	- 	+	+	Bacillus Spp	
28.		2PS1B1	Gray 	Circular 	Smooth 	Raised 	Medium 	Rod	+	+	+	-	-	+	+	+	- 	+	+	Bacillus Spp	
29.		2PS2B3	Creamy 	Circular 	Smooth 	Flat	Large 	Rod	+	+	+	-	-	+	+	+	- 	+	-	Bacillus Spp	
30.		2PS3B2	Creamy 	Circular 	Smooth 	Flat	Large 	Rod	+	+	+	-	-	+	+	+	- 	+	-	Bacillus Spp	
31.		2PS4B2	White  	Circular 	Rough  	Raised 	Large 	Rod	+	+	+	-	-	+	+	+	- 	+	+	Bacillus Spp	
32.		2BK1B1	White	Circular 	Smooth 	Raised 	Small 	Coccus 	+	-	-	-	-	+	+	+	-	+	+	Staphylococcus Spp. 	
33.		2BM2B2	White	Circular 	Smooth 	Raised 	Small 	Coccus 	+	-	-	-	-	+	+	+	+	-	-	Staphylococcus Spp	
34.		2BK3B1	White	Circular 	Smooth 	Raised 	Small 	Coccus 	+	-	-	-	-	+	+	+	+	-	-	Staphylococcus Spp	
35.		2BK4B2	White	Circular 	Smooth 	Raised 	Small 	Coccus 	+	-	-	-	-	+	+	+	+	-	-	Staphylococcus Spp. 	
36.		2BM1B4	White	Circular 	Smooth 	Raised 	Small 	Coccus 	+	-	-	-	-	+	+	+	-	+	+	Staphylococcus Spp	
37.		2BM4B1	White	Circular 	Smooth 	Raised 	Small 	Coccus 	+	-	-	-	-	+	+	+	+	-	-	Staphylococcus Spp	
38.		2BS2B4	White	Circular 	Smooth 	Raised 	Small 	Coccus 	+	-	-	-	-	+	+	+	+	-	-	Staphylococcus Spp. 	
39.		2BS3B2	White	Circular 	Smooth 	Raised 	Small 	Coccus 	+	-	-	-	-	+	+	+	+	-	-	Staphylococcus Spp	
40.		2OM1B3	White	Circular 	Smooth 	Raised 	Small 	Coccus 	+	-	-	-	-	+	+	+	+	-	-	Staphylococcus Spp	
41.		2OK2B3	White	Circular 	Smooth 	Raised 	Small 	Coccus 	+	-	-	-	-	+	+	+	-	+	+	Staphylococcus Spp	
42.		2OK3B2	White	Circular 	Smooth 	Raised 	Small 	Coccus 	+	-	-	-	-	+	+	+	+	-	-	Staphylococcus Spp	
43.		2OM4B3	White	Circular 	Smooth 	Raised 	Small 	Coccus 	+	-	-	-	-	+	+	+	+	-	-	Staphylococcus Spp	
44.		2OS3B2	White	Circular 	Smooth 	Raised 	Small 	Coccus 	+	-	-	-	-	+	+	+	+	-	-	Staphylococcus Spp	
45.		2PK1B4	White	Circular 	Smooth 	Raised 	Small 	Coccus 	+	-	-	-	-	+	+	+	+	-	-	Staphylococcus Spp	
46.		2PK2B2	White	Circular 	Smooth 	Raised 	Small 	Coccus 	+	-	-	-	-	+	+	+	+	-	-	Staphylococcus Spp	
47.		2PM3B1	White	Circular 	Smooth 	Raised 	Small 	Coccus 	+	-	-	-	-	+	+	+	+	-	-	Staphylococcus Spp	
48.		2PM4B2	White	Circular 	Smooth 	Raised 	Small 	Coccus 	+	-	-	-	-	+	+	+	-	+	+	Staphylococcus Spp. 	
49.		2PM1B1	White	Circular 	Smooth 	Raised 	Small 	Coccus 	+	-	-	-	-	+	+	+	+	-	-	Staphylococcus Spp	
50.		2PS1B3	White	Circular 	Smooth 	Raised 	Small 	Coccus 	+	-	-	-	-	+	+	+	-	+	+	Staphylococcus Spp	
51.		2PS3B2	White	Circular 	Smooth 	Raised 	Small 	Coccus 	+	-	-	-	-	+	+	+	-	+	+	Staphylococcus Spp	
52.		2PS4B3	White	Circular 	Smooth 	Raised 	Small 	Coccus 	+	-	-	-	-	+	+	+	+	-	-	Staphylococcus Spp	
53.		2BK3B1	White	Circular 	Smooth 	Raised 	Small 	Rod	-	+	-	+	+	-	+	+	-	+	+	Pseudomonas Spp	
54.		2BM1B1	White	Circular 	Smooth 	Raised 	Small 	Rod	-	+	-	+	+	-	+	+	-	+	-	Pseudomonas Spp	
55.		2BM3B2	White	Circular 	Smooth 	Raised 	Small 	Rod	-	+	-	+	+	-	+	+	-	+	+	Pseudomonas Spp	
56.		2BS3B5	White	Circular 	Smooth 	Raised 	Small 	Rod	-	+	-	-	+	-	+	+	-	+	-	Pseudomonas Spp	
57.		2OK1B2	White	Circular 	Smooth 	Raised 	Small 	Rod	-	+	-	-	+	-	+	+	-	+	-	Pseudomonas Spp	
58.		2OK2B1	White	Circular 	Smooth 	Raised 	Small 	Rod	-	+	-	-	+	-	+	+	-	+	-	Pseudomonas Spp	
59.		2OM1B2	White	Circular 	Smooth 	Raised 	Small 	Rod	-	+	-	+	+	-	+	+	-	+	-	Pseudomonas Spp	
60.		2OM3B4	White	Circular 	Smooth 	Raised 	Small 	Rod	-	+	-	-	+	-	+	+	-	+	+	Pseudomonas Spp	
61.		2OM4B2	White	Circular 	Smooth 	Raised 	Small 	Rod	-	+	-	-	+	-	+	+	-	+	-	Pseudomonas Spp	
62.		2OS2B2	White	Circular 	Smooth 	Raised 	Small 	Rod	-	+	-	-	+	-	+	+	-	+	-	Pseudomonas Spp	
63.		2OS3B1	White	Circular 	Smooth 	Raised 	Small 	Rod	-	+	-	+	+	-	+	+	-	+	-	Pseudomonas Spp	
64.		2BK1B3	Gray  	Circular 	Smooth 	Raised 	Medium 	Rod	-	+	-	+	+	-	+	-	-	+	-	E. coli	
65.		2BM2B1	Creamy 	Circular 	Smooth 	Raised 	Medium 	Rod	-	+	-	+	+	-	+	-	-	+	-	E. coli	
66.		2BM3B1	Creamy 	Circular 	Smooth 	Raised 	Medium 	Rod	-	+	-	+	+	-	+	-	-	+	-	E. coli	
67.		2BS3B3	Creamy 	Circular 	Smooth 	Raised 	Medium 	Rod	-	+	-	+	+	-	+	-	-	+	-	E. coli	
68.		2OM3B2	Gray  	Circular 	Smooth 	Raised 	Medium 	Rod	-	+	-	+	+	-	+	-	-	+	-	E. coli	
69.		2OS1B1	Creamy 	Circular 	Smooth 	Raised 	Medium 	Rod	-	+	-	+	+	-	+	-	-	+	-	E. coli	
57.		2PM1B1	Gray  	Circular 	Smooth 	Raised 	Medium 	Rod	-	+	-	+	+	-	+	-	-	+	-	E. coli	
58.		2PM2B3	Creamy 	Circular 	Smooth 	Raised 	Medium 	Rod	-	+	-	+	+	-	+	-	-	+	-	E. coli	
59.		2PS3B1	Creamy 	Circular 	Smooth 	Raised 	Medium 	Rod	-	+	-	+	+	-	+	-	-	+	-	E. coli	
60.		2BK2B3	Creamy 	Circular 	Smooth 	Raised 	Medium 	Rod	-	+	-	-	+	+	+	+	-	+	-	Salmonella Spp	
61.		2BM2B3	Creamy 	Circular 	Smooth 	Raised 	Medium 	Rod	-	+	-	-	+	+	+	+	-	+	-	Salmonella Spp	
62.		2PM3B2	Creamy 	Circular 	Smooth 	Raised 	Medium 	Rod	-	+	-	-	+	+	+	+	-	+	-	Salmonella Spp	
63.		2PM4B3	Creamy 	Circular 	Smooth 	Raised 	Medium 	Rod	-	+	-	-	+	+	+	+	-	+	-	Salmonella Spp	
64.		2PS2B2	Creamy 	Circular 	Smooth 	Raised 	Medium 	Rod	-	+	-	-	+	+	+	+	-	+	-	Salmonella Spp	
65.		2BM1B2	Creamy 	Circular 	Smooth 	Raised 	Medium 	Rod	-	-	-	-	-	+	+	-	-	+	-	Shigella Spp	
66.		2PK2B4	Creamy 	Circular 	Smooth 	Raised 	Medium 	Rod	-	-	-	-	-	+	+	-	-	+	-	Shigella Spp	
67.		2PM1B3	Creamy 	Circular 	Smooth 	Raised 	Medium 	Rod	-	-	-	-	-	+	+	-	-	+	-	Shigella Spp	
68.		2BM1B1	Red 	Circular 	Smooth 	Raised 	Medium 	Rod	-	+	-	-	-	+	+	-	-	-	-	Serratia Spp	
69.		2PS3B1	Red 	Circular 	Smooth 	Raised 	Medium 	Rod	-	+	-	-	-	+	+	-	-	-	-	Serratia Spp	

Table 8. Morphological and biochemical characterization of yeast isolates from moderately ripened fruit samples
No	
Isolate Code	Colony Morphology on SDA	
Colony Color on BiGGY. Agar	
Germ Tube 	
Presumptive  Species	
		Color	Shape 	Size	Texture 	Elevation				
1.		2BK1Y1	Creamy 	Circular	Medium 	Smooth	Convex	Light Brown	-	Candida Spp.	
2.		2BK1Y2	White 	Circular	Medium 	Rough	Raised 	Creamy 	-	Saccharomyces Spp 	
3.		2BK2Y1	White 	Circular	Medium 	Smooth	Raised 	Creamy 	-	Saccharomyces Spp 	
4.		2BK2Y2	Creamy 	Circular	Small 	Smooth	Convex	Light Brown	-	Candida Spp.	
5.		2BK3Y1	Creamy 	Circular	Medium 	Smooth	Convex	Dark brown/yellow halo	-	Candida Spp.	
6.		2BK3Y2	Creamy 	Circular	Medium 	Smooth	Convex	Brown/black center	-	Candida Spp.	
7.		2BK3Y3	Creamy 	Circular	Large 	Smooth	Flat	Dark brown/yellow halo	+	Candida albican	
8.		2BK4Y2	White 	Circular	Small 	Rough 	Raised 	No Growth	-	Saccharomyces Spp 	
9.		2BK4Y3	Creamy 	Circular	Medium 	Smooth	Convex	Brown/black center	-	Candida Spp.	
10.		2BM1Y1	Creamy 	Circular	Large 	Smooth	Flat 	Dark brown/yellow halo	+	Candida albican	
11.		2BM1Y2	White 	Circular	Small 	Smooth	Convex	Creamy 	-	Saccharomyces Spp 	
12.		2BM1Y2	Creamy 	Circular	Small 	Smooth	Convex	Dark brown/yellow halo	-	Candida Spp.	
13.		2BM2Y3	Creamy 	Circular	Medium 	Smooth	Convex	Brown/black center	-	Candida Spp.	
14.		2BM2Y4	White 	Circular	Small 	Rough 	Raised 	Creamy 	-	Saccharomyces Spp 	
15.		2BM3Y1	White 	Circular	Medium 	Smooth	Raised 	Creamy 	-	Saccharomyces Spp 	
16.		2BM3Y4	Creamy 	Circular	Medium 	Smooth	Convex	Light Brown	-	Candida Spp.	
17.		2BS1Y3	White 	Circular	Medium 	Smooth	Convex	Creamy 	-	Saccharomyces Spp 	
18.		2BS2Y2	Creamy 	Circular	Large 	Smooth	Flat	Dark brown/yellow halo	-	Candida Spp.	
19.		2BS2Y3	White 	Circular	Medium 	Smooth	Raised 	Creamy 	-	Saccharomyces Spp 	
20.		2BS3Y1	Creamy 	Circular	Large 	Smooth	Flat 	Dark brown/yellow halo	-	Candida Spp.	
21.		2BS3Y2	Creamy 	Circular	Medium 	Smooth	Convex	Dark brown/yellow halo	-	Candida Spp.	
22.		2BS4Y3	Creamy 	Circular	Large 	Smooth	Convex	Brown/black center	-	Candida Spp.	
23.		2OK1Y1	Creamy 	Circular	Large 	Smooth	Convex	Dark brown/yellow halo	+	Candida albican	
24.		2OK1Y2	Creamy 	Circular	Large 	Smooth	Flat	Dark brown/yellow halo	-	Candida Spp.	
25.		2OK1Y3	White 	Circular	Small 	Rough 	Raised 	Creamy 	-	Saccharomyces Spp 	
26.		2OK2Y1	Creamy 	Circular	Medium 	Smooth	Convex	Brown/black center	-	Candida Spp.	
27.		2OK2Y2	White 	Circular	Medium 	Smooth	Convex	No Growth	-	Saccharomyces Spp 	
28.		2OK2Y3	Creamy 	Circular	Large 	Smooth	Flat	Dark brown/yellow halo	+	Candida albican	
29.		2OM1Y2	Creamy 	Circular	Medium 	Smooth	Convex	Dark brown/yellow halo	-	Candida Spp.	
30.		2OM2Y1	White 	Circular	Medium 	Smooth	Raised 	Creamy 	-	Saccharomyces Spp 	
31.		2OM2Y3	Creamy 	Circular	Large 	Smooth	Convex	Light Brown	-	Candida Spp.	
32.		2OM3Y1	Creamy 	Circular	Large 	Smooth	Flat	Dark brown/yellow halo	-	Candida Spp.	
33.		2OM3Y2	White 	Circular	Medium 	Smooth	Raised 	No Growth	-	Saccharomyces Spp 	
34.		2OM4Y2	Creamy 	Circular	Large 	Smooth	Convex	Light Brown	-	Candida Spp.	
35.		2OS1Y2	White 	Circular	Medium 	Smooth	Convex	No Growth	-	Saccharomyces Spp 	
36.		2OS1Y3	Creamy 	Circular	Small 	Smooth	Convex	Brown/black center	-	Candida Spp.	
37.		2OS2Y1	Creamy 	Circular	Large 	Smooth	Flat 	Dark brown/yellow halo	-	Candida Spp.	
38.		2OS3Y1	Creamy 	Circular	Large 	Smooth	Convex	Brown/black center	-	Candida Spp.	
39.		2OS3Y2	White 	Circular	Medium 	Smooth	Convex	Creamy 	-	Saccharomyces Spp 	
40.		2OS4Y1	Creamy 	Circular	Large	Smooth	Flat	Brown/black center	-	Candida Spp.	
41.		2OS4Y2	Creamy 	Circular	Medium 	Smooth	Convex	Dark brown/yellow halo	-	Candida Spp.	
42.		2PK1Y1	White 	Circular	Medium 	Rough 	Raised 	No Growth	-	Saccharomyces Spp 	
43.		2PK1Y2	Creamy 	Circular	Medium 	Smooth	Convex	Brown/black center	-	Candida Spp.	
44.		2PK2Y3	Creamy 	Circular	Small 	Smooth	Convex	Brown/black center	-	Candida Spp.	
45.		2PK2Y4	White 	Circular	Small 	Smooth	Raised 	Creamy 	-	Saccharomyces Spp 	
46.		2PK3Y1	Creamy 	Circular	Medium 	Smooth	Convex	Dark brown/yellow halo	-	Candida Spp.	
47.		2PK4Y1	Creamy 	Circular	Large 	Smooth	Convex	Brown/black center	-	Candida Spp.	
48.		2PK4Y2	White 	Circular	Small 	Smooth	Raised 	Creamy 	-	Saccharomyces Spp 	
49.		2PM1Y1	Creamy 	Circular	Large 	Smooth	Flat	Black/yellow halo	+	Candida albican	
50.		2PM1Y3	White 	Circular	Small 	Smooth	Raised 	Creamy 		Saccharomyces Spp 	
51.		2PM1Y4	Creamy 	Circular	Small 	Smooth	Convex	Brown/black center	-	Candida Spp.	
52.		2PM2Y1	Creamy 	Circular	Medium 	Smooth	Convex	Black/yellow halo	+	Candida albican	
53.		2PM2Y2	White 	Circular	Medium 	Rough 	Raised 	No Growth	-	Saccharomyces Spp 	
54.		2PM2Y3	Creamy 	Circular	Medium 	Smooth	Convex	Black/yellow halo	-	Candida Spp.	
55.		2PM3Y1	Creamy 	Circular	Small 	Smooth	Convex	Dark brown/yellow halo	-	Candida Spp.	
56.		2PM3Y2	Creamy 	Circular	Medium 	Smooth	Convex	Dark brown/yellow halo	+	Candida albican	
57.		2PM3Y3	White 	Circular	Small 	Smooth	Raised 	Creamy 	-	Saccharomyces Spp 	
58.		2PS1Y1	Creamy 	Circular	Medium 	Smooth	Convex	Brown/black center	-	Candida Spp.	
59.		2PS1Y3	White 	Circular	Small 	Rough 	Raised 	Creamy 		Saccharomyces Spp 	
60.		2PS2Y2	Creamy 	Circular	Medium 	Smooth	Convex	Brown/black center	+	Candida albican	
61.		2PS2Y3	Creamy 	Circular	Small 	Smooth	Convex	Brown/black center	-	Candida Spp.	
62.		2PS2Y4	White 	Circular	Medium 	Smooth	Raised 	No Growth	-	Saccharomyces Spp 	
63.		2PS3Y1	Creamy 	Circular	Medium 	Smooth	Convex 	Dark brown/yellow halo	-	Candida Spp.	
64.		2PS3Y2	Creamy 	Circular	Small 	Smooth	Convex	Light Brown	+	Candida albican	
65.		2PS4Y2	White 	Circular	Small 	Rough 	Raised 	Creamy 	-	Saccharomyces Spp 	
66.		2PS4Y3	Creamy 	Circular	Large 	Smooth	Flat 	Black/yellow halo	-	Candida Spp.	


Table 9. Morphological characterization of molds isolates from moderately ripened fruit samples grown on PDA
No	Isolate Code	Front color	Reverse side  color	Growth Pattern	Presumptive  Species	
1.		2BK1M3	Greenish gray 	Creamy	Dominant	Fusarium Spp.	
2.		2BK2M2	Black 	Creamy 	Dominant 	Aspergillus Spp.	
3.		2BK3M3	Black 	Creamy 	Dominant 	Fusarium Spp.	
4.		2BK4M1	Black 	Creamy 	Dominant 	Aspergillus Spp.	
5.		2BM1M1	Black 	Black 	Dominant  	Alternaria Spp.	
6.		2BM1M2	Black 	Creamy 	Dominant 	Aspergillus Spp.	
7.		2BM1M4	Black 	Creamy 	Dominant 	Fusarium Spp.	
8.		2BM2M2	Black 	Creamy 	Dominant 	Aspergillus Spp.	
9.		2BM2M3	Black 	Balck 	Dominant  	Fusarium Spp.	
10.		2BM3M1	Black 	Creamy 	Dominant 	Aspergillus Spp.	
11.		2BS1M2	Black 	Creamy 	Dominant 	Aspergillus Spp.	
12.		2BS2M3	Black 	Black 	Dominant  	Alternaria Spp.	
13.		2BS3M1	Black 	Creamy 	Dominant 	Aspergillus Spp.	
14.		2BS3M3	Greenish gray 	Creamy	Dominant	Penicellium Spp.	
15.		2BS43M2	Black 	Creamy 	Dominant 	Aspergillus Spp.	
16.		2OK1M1	Black 	Creamy 	Dominant 	Aspergillus Spp.	
17.		2OK1M5	White 	Light yellow	Poor 	Botirytis Spp.	
18.		2OK2M3	Black 	Creamy 	Dominant 	Aspergillus Spp.	
19.		2OK3M1	Black 	Black 	Dominant  	Alternaria Spp.	
20.		2OK3M2	Greenish gray 	Creamy	Dominant	Penicellium Spp.	
21.		2OK4M1	Greenish gray 	Creamy	Dominant	Penicellium Spp.	
22.		2OM1M1	Dark gray	Creamy	Poor 	Aspergillus Spp.	
23.		2OM1M2	Greenish gray 	Creamy	Dominant	Penicellium Spp.	
24.		2OM3M1	Black 	Creamy 	Dominant 	Penicellium Spp.	
25.		2OM3M3	Black 	Black 	Dominant  	Alternaria Spp.	
26.		2OM4M2	Greenish gray 	Creamy	Dominant	Fusarium Spp.	
27.		2OS1M2	Black 	Black 	Dominant  	Alternaria Spp.	
28.		2OS2M2	Dark gray	Creamy	Poor 	Aspergillus Spp.	
29.		2OS3M1	Dark gray	Creamy	Poor 	Aspergillus Spp.	
30.		2OS3M3	Black 	Black 	Dominant	Alternaria Spp.	
31.		2OS3M4	White 	Light yellow	Poor 	Botirytis Spp.	
32.		2PK1M1	Dark gray	Creamy	Poor 	Aspergillus Spp.	
33.		2PK1M2	White 	Light yellow	Dominant 	Rhizopus Spp	
34.		2PK2M1	Black 	Black 	Dominant  	Alternaria Spp.	
35.		2PK2M2	Black 	Creamy 	Dominant 	Aspergillus Spp.	
36.		2PM1M3	Black 	Creamy 	Dominant 	Aspergillus Spp.	
37.		2PM2M1	White 	Light yellow	Dominant 	Rhizopus Spp.	
38.		2PM2M2	Black 	Black 	Dominant  	Alternaria Spp.	
39.		2PM3M2	Black 	Creamy 	Dominant 	Aspergillus Spp.	
40.		2PM4M1	Greenish gray 	Creamy	Dominant	Penicellium Spp.	
41.		2PM4M3	Black 	Creamy 	Dominant 	Aspergillus Spp.	
42.		2PS1M1	Black 	Creamy 	Dominant 	Penicellium Spp.	
43.		2PS1M3	White 	Light yellow	Dominant 	Aspergillus Spp.	
44.		2PS1M4	Black 	Creamy 	Dominant 	Fusarium Spp.	
45.		2PS2M1	Greenish gray 	Creamy	Dominant	Penicellium Spp.	
46.		2PS2M2	Black 	Creamy 	Dominant 	Aspergillus Spp.	
47.		2PS2M3	Greenish gray 	Creamy	Dominant	Fusarium Spp.	
48.		2PS3M1	Black 	Black 	Dominant  	Alternaria Spp.	
49.		2PS3M2	Dark gray 	Creamy 	Poor 	Aspergillus Spp.	
50.		2PS3M3	Black 	Creamy 	Dominant 	Fusarium Spp.	
Table 10. Morphological and biochemical characterization of bacterial isolates from overripe fruit samples
No	Isolate/ Code	Colony Morphology on NA	

Cell Shape	Gram reaction	Motility	Endospore 	Indole	H2S	TSIA	Catalase	Citrate	Coagulase 	Oxidase 	Urease	Presumptive  Species	
		Color 	Shape	Text.	Elev. 	Size 														
1.		3BK1B1	Creamy 	Circular 	Smooth 	Flat	Medium 	Rod	+	+	+	-	-	+	+	+	- 	+	-	Bacillus Spp	
2.		3BK2B2	Creamy 	Circular 	Smooth 	Flat	Large 	Rod 	+	+	+	-	-	+	+	+	- 	+	-	Bacillus Spp	
3.		3BK3B2	Gray 	Circular 	Smooth 	Flat	Medium 	Rod	+	+	+	-	-	+	+	+	- 	+	+	Bacillus Spp	
4.		3BK4B1	Creamy 	Circular 	Smooth 	Flat	Medium 	Rod	+	+	+	-	-	+	+	+	- 	+	-	Bacillus Spp	
5.		3BM1B1 	White 	Circular 	Rough  	Raised 	Large 	Rod	+	+	+	-	-	+	+	+	- 	+	+	Bacillus Spp	
6.		3BM2B2	Creamy 	Circular 	Smooth 	Flat	Medium 	Rod	+	+	+	-	-	+	+	+	- 	+	-	Bacillus Spp	
7.		3BM3B2	Creamy 	Circular 	Smooth 	Flat 	Medium 	Rod	+	+	+	-	-	+	+	+	- 	+	-	Bacillus Spp	
8.		3BM4B2	White 	Circular 	Rough  	Raised	Large 	Rod	+	+	+	-	-	+	+	+	- 	+	+	Bacillus Spp	
9.		3BM1B3	Creamy 	Circular 	Smooth 	Flat	Medium 	Rod	+	+	+	-	-	+	+	+	- 	+	-	Bacillus Spp	
10.		3BM4B1	Creamy 	Circular 	Smooth 	Flat	Medium 	Rod	+	+	+	-	-	+	+	+	- 	+	-	Bacillus Spp	
11.		3BK1B2	Creamy 	Circular 	Smooth 	Flat	Medium 	Rod	+	+	+	-	-	+	+	+	- 	+	-	Bacillus Spp	
12.		3BK2B1	Creamy 	Circular 	Smooth 	Flat	Medium 	Rod	+	+	+	-	-	+	+	+	- 	+	-	Bacillus Spp	
13.		3BK3B2	Gray 	Circular 	Smooth 	Flat	Large 	Rod	+	+	+	-	-	+	+	+	- 	+	+	Bacillus Spp	
14.		3BK4B2	Creamy 	Circular 	Smooth 	Flat	Large 	Rod	+	+	+	-	-	+	+	+	- 	+	-	Bacillus Spp	
15.		3OK1B3	Creamy 	Circular 	Smooth 	Flat	Medium 	Rod	+	+	+	-	-	+	+	+	- 	+	-	Bacillus Spp	
16.		3OK2B1	Gray 	Circular 	Smooth 	Flat	Medium 	Rod	+	+	+	-	-	+	+	+	- 	+	+	Bacillus Spp	
17.		3OM1B2	Creamy 	Circular 	Smooth 	Flat	Medium 	Rod	+	+	+	-	-	+	+	+	- 	+	-	Bacillus Spp	
18.		3OM3B3	Gray 	Circular 	Smooth 	Flat	Medium 	Rod	+	+	+	-	-	+	+	+	- 	+	+	Bacillus Spp	
19.		3OM2B2	Creamy 	Circular 	Smooth 	Flat	Medium 	Rod	+	+	+	-	-	+	+	+	- 	+	-	Bacillus Spp	
20.		3OM2B3	Creamy 	Circular 	Smooth 	Flat	Medium 	Rod	+	+	+	-	-	+	+	+	- 	+	-	Bacillus Spp	
21.		3OM4B2	Gray 	Circular 	Smooth 	Flat	Medium 	Rod	+	+	+	-	-	+	+	+	- 	+	+	Bacillus Spp	
22.		3OS1B1	Creamy 	Circular 	Smooth 	Flat	Large 	Rod	+	+	+	-	-	+	+	+	- 	+	-	Bacillus Spp	
23.		3OS1B2	Creamy 	Circular 	Smooth 	Flat	Large 	Rod	+	+	+	-	-	+	+	+	- 	+	-	Bacillus Spp	
24.		3OS2B1	Creamy 	Circular 	Smooth 	Flat	Large 	Rod	+	+	+	-	-	+	+	+	- 	+	-	Bacillus Spp	
25.		3OS3B2	Gray 	Circular 	Smooth 	Raised 	Medium 	Rod	+	+	+	-	-	+	+	+	- 	+	+	Bacillus Spp	
26.		3PK1B1	Gray 	Circular 	Smooth 	Raised 	Medium 	Rod	+	+	+	-	-	+	+	+	- 	+	+	Bacillus Spp	
27.		3PK2B2	Creamy 	Circular 	Smooth 	Flat 	Medium 	Rod	+	+	+	-	-	+	+	+	- 	+	-	Bacillus Spp	
28.		3PK3B2	Gray 	Circular 	Smooth 	Raised 	Medium 	Rod	+	+	+	-	-	+	+	+	- 	+	+	Bacillus Spp	
29.		3PK4B1	Creamy 	Circular 	Smooth 	Flat	Large 	Rod	+	+	+	-	-	+	+	+	- 	+	-	Bacillus Spp	
30.		3PM1B2	Creamy 	Circular 	Smooth 	Flat	Large 	Rod	+	+	+	-	-	+	+	+	- 	+	-	Bacillus Spp	
31.		3PM2B5	Gray 	Circular 	Smooth 	Raised 	Medium 	Rod	+	+	+	-	-	+	+	+	- 	+	+	Bacillus Spp	
32.		3PM3B1	Creamy 	Circular 	Smooth 	Flat	Medium 	Rod	+	+	+	-	-	+	+	+	- 	+	-	Bacillus Spp	
33.		3PM4B2	Creamy 	Circular 	Smooth 	Flat	Medium 	Rod	+	+	+	-	-	+	+	+	- 	+	-	Bacillus Spp	
34.		3PM2B4	White 	Circular 	Rough  	Raised 	Large 	Rod	+	+	+	-	-	+	+	+	- 	+	-	Bacillus Spp	
35.		3PM3B1	White 	Circular 	Rough  	Raised 	Large 	Rod	+	+	+	-	-	+	+	+	- 	+	+	Bacillus Spp	
36.		3PM3B3	Creamy 	Circular 	Smooth 	Flat	Medium 	Rod	+	+	+	-	-	+	+	+	- 	+	-	Bacillus Spp	
37.		3PM2B1	Creamy 	Circular 	Smooth 	Flat	Medium 	Rod	+	+	+	-	-	+	+	+	- 	+	-	Bacillus spp	
38.		3PS1B1	Creamy 	Circular 	Smooth 	Flat	Medium 	Rod	+	+	+	-	-	+	+	+	- 	+	-	Bacillus Spp	
39.		3PS1B2	White 	Circular 	Rough  	Raised 	Large 	Rod	+	+	+	-	-	+	+	+	- 	+	+	Bacillus Spp	
40.		3PS2B3	Creamy 	Circular 	Smooth 	Flat	Medium 	Rod	+	+	+	-	-	+	+	+	- 	+	-	Bacillus Spp	
41.		3PS3B1	Creamy 	Circular 	Smooth 	Flat	Medium 	Rod	+	+	+	-	-	+	+	+	- 	+	-	Bacillus Spp	
42.		3BK1B3	White	Circular 	Smooth 	Raised 	Small 	Coccus 	+	-	-	-	-	+	+	+	+	-	-	Staphylococcus Spp	
43.		3BK2B4	White	Circular 	Smooth 	Raised 	Small 	Coccus 	+	-	-	-	-	+	+	+	+	-	-	Staphylococcus Spp	
44.		3BK4B4	White	Circular 	Smooth 	Raised 	Small 	Coccus 	+	-	-	-	-	+	+	+	+	-	-	Staphylococcus Spp	
45.		3BM2B3	White	Circular 	Smooth 	Raised 	Small 	Coccus 	+	-	-	-	-	+	+	+	-	+	+	Staphylococcus Spp	
46.		3BM3B3	White	Circular 	Smooth 	Raised 	Small 	Coccus 	+	-	-	-	-	+	+	+	+	-	-	Staphylococcus Spp	
47.		3BM4B2	White	Circular 	Smooth 	Raised 	Small 	Coccus 	+	-	-	-	-	+	+	+	-	+	+	Staphylococcus Spp	
48.		3BS1B4	White	Circular 	Smooth 	Raised 	Small 	Coccus 	+	-	-	-	-	+	+	+	-	+	+	Staphylococcus Spp	
49.		3BS2B1	White	Circular 	Smooth 	Raised 	Small 	Coccus 	+	-	-	-	-	+	+	+	+	-	-	Staphylococcus Spp	
50.		3BS3B1	White	Circular 	Smooth 	Raised 	Small 	Coccus 	+	-	-	-	-	+	+	+	+	-	-	Staphylococcus Spp	
51.		3OK2B3	White	Circular 	Smooth 	Raised 	Small 	Coccus 	+	-	-	-	-	+	+	+	-	+	+	Staphylococcus Spp	
52.		3OK3B1	White	Circular 	Smooth 	Raised 	Small 	Coccus 	+	-	-	-	-	+	+	+	+	-	-	Staphylococcus Spp	
53.		3OM1B1	White	Circular 	Smooth 	Raised 	Small 	Coccus 	+	-	-	-	-	+	+	+	+	-	-	Staphylococcus Spp. 	
54.		3OM2B1	White	Circular 	Smooth 	Raised 	Small 	Coccus 	+	-	-	-	-	+	+	+	-	+	+	Staphylococcus Spp	
55.		3OM3B1	White	Circular 	Smooth 	Raised 	Small 	Coccus 	+	-	-	-	-	+	+	+	+	-	-	Staphylococcus Spp. 	
56.		3OS2B3	White	Circular 	Smooth 	Raised 	Small 	Coccus 	+	-	-	-	-	+	+	+	-	+	+	Staphylococcus Spp	
57.		3OS4B3	White	Circular 	Smooth 	Raised 	Small 	Coccus 	+	-	-	-	-	+	+	+	+	-	-	Staphylococcus Spp	
58.		3PK1B2	White	Circular 	Smooth 	Raised 	Small 	Coccus 	+	-	-	-	-	+	+	+	-	+	+	Staphylococcus Spp	
59.		3PK2B3	White	Circular 	Smooth 	Raised 	Small 	Coccus 	+	-	-	-	-	+	+	+	+	-	-	Staphylococcus Spp	
60.		3PK3B3	White	Circular 	Smooth 	Raised 	Small 	Coccus 	+	-	-	-	-	+	+	+	-	+	+	Staphylococcus Spp	
61.		3PK4B4	White	Circular 	Smooth 	Raised 	Small 	Coccus 	+	-	-	-	-	+	+	+	+	-	-	Staphylococcus Spp	
62.		3PM1B4	White	Circular 	Smooth 	Raised 	Small 	Coccus 	+	-	-	-	-	+	+	+	-	+	+	Staphylococcus Spp. 	
63.		3PM2B3	White	Circular 	Smooth 	Raised 	Small 	Coccus 	+	-	-	-	-	+	+	+	+	-	-	Staphylococcus Spp	
64.		3PM3B5	White	Circular 	Smooth 	Raised 	Small 	Coccus 	+	-	-	-	-	+	+	+	+	-	-	Staphylococcus Spp	
65.		3PM4B2	White	Circular 	Smooth 	Raised 	Small 	Coccus 	+	-	-	-	-	+	+	+	-	+	+	Staphylococcus Spp	
66.		3PS1B2	White	Circular 	Smooth 	Raised 	Small 	Coccus 	+	-	-	-	-	+	+	+	-	+	+	Staphylococcus Spp	
67.		3PS2B4	White	Circular 	Smooth 	Raised 	Small 	Coccus 	+	-	-	-	-	+	+	+	+	-	-	Staphylococcus Spp	
68.		3PS3B1	White	Circular 	Smooth 	Raised 	Small 	Coccus 	+	-	-	-	-	+	+	+	+	-	-	Staphylococcus Spp	
69.		2BK4B2	White	Circular 	Smooth 	Raised 	Small 	Rod	-	+	-	+	+	-	+	+	-	+	-	Pseudomonas Spp	
70.		2BK2B4	White	Circular 	Smooth 	Raised 	Small 	Rod	-	+	-	-	+	-	+	+	-	+	+	Pseudomonas Spp	
71.		2BM3B5	White	Circular 	Smooth 	Raised 	Small 	Rod	-	+	-	+	+	-	+	+	-	+	+	Pseudomonas Spp	
72.		2BM2B3	White	Circular 	Smooth 	Raised 	Small 	Rod	-	+	-	-	+	-	+	+	-	+	-	Pseudomonas Spp	
73.		2BS3B3	White	Circular 	Smooth 	Raised 	Small 	Rod	-	+	-	-	+	-	+	+	-	+	-	Pseudomonas Spp	
74.		2OS4B1	White	Circular 	Smooth 	Raised 	Small 	Rod	-	+	-	-	+	-	+	+	-	+	-	Pseudomonas Spp	
75.		2OK1B3	White	Circular 	Smooth 	Raised 	Small 	Rod	-	+	-	+	+	-	+	+	-	+	-	Pseudomonas Spp	
76.		2OK2B3	White	Circular 	Smooth 	Raised 	Small 	Rod	-	+	-	-	+	-	+	+	-	+	+	Pseudomonas Spp	
77.		2OK3B2	White	Circular 	Smooth 	Raised 	Small 	Rod	-	+	-	-	+	-	+	+	-	+	-	Pseudomonas Spp	
78.		2OM2B1	White	Circular 	Smooth 	Raised 	Small 	Rod	-	+	-	-	+	-	+	+	-	+	-	Pseudomonas Spp	
79.		2OM3B1	White	Circular 	Smooth 	Raised 	Small 	Rod	-	+	-	-	+	-	+	+	-	+	+	Pseudomonas Spp	
80.		2OM4B1	White	Circular 	Smooth 	Raised 	Small 	Rod	-	+	-	+	+	-	+	+	-	+	+	Pseudomonas Spp	
81.		2OS1B3	White	Circular 	Smooth 	Raised 	Small 	Rod	-	+	-	-	+	-	+	+	-	+	-	Pseudomonas Spp	
82.		2PS4B1	White	Circular 	Smooth 	Raised 	Small 	Rod	-	+	-	+	+	-	+	+	-	+	-	Pseudomonas Spp	
83.		2PK1B4	White	Circular 	Smooth 	Raised 	Small 	Rod	-	+	-	-	+	-	+	+	-	+	-	Pseudomonas Spp	
84.		2PK2B3	White	Circular 	Smooth 	Raised 	Small 	Rod	-	+	-	+	+	-	+	+	-	+	-	Pseudomonas Spp	
85.		2PM3B1	White	Circular 	Smooth 	Raised 	Small 	Rod	-	+	-	-	+	-	+	+	-	+	+	Pseudomonas Spp	
86.		2PM4B3	White	Circular 	Smooth 	Raised 	Small 	Rod	-	+	-	-	+	-	+	+	-	+	+	Pseudomonas Spp	
87.		3BK4B3	Creamy 	Circular 	Smooth 	Raised 	Medium 	Rod	-	+	-	+	+	-	+	-	-	+	-	E. coli 	
88.		3BK2B5	Creamy 	Circular 	Smooth 	Raised 	Medium 	Rod	-	+	-	+	+	-	+	-	-	+	-	E. coli 	
89.		3BM1B3	Creamy 	Circular 	Smooth 	Raised 	Medium 	Rod	-	+	-	+	+	-	+	-	-	+	-	E. coli 	
90.		3BM2B4	Gray  	Circular 	Smooth 	Raised 	Medium 	Rod	-	+	-	+	+	-	+	-	-	+	-	E. coli 	
91.		3BS4B4	Creamy 	Circular 	Smooth 	Raised 	Medium 	Rod	-	+	-	+	+	-	+	-	-	+	-	E. coli 	
92.		3OK2B2	Creamy 	Circular 	Smooth 	Raised 	Medium 	Rod	-	+	-	+	+	-	+	-	-	+	-	E. coli 	
93.		3OK3B5	Gray  	Circular 	Smooth 	Raised 	Medium 	Rod	-	+	-	+	+	-	+	-	-	+	-	E. coli 	
94.		3OS3B3	Gray  	Circular 	Smooth 	Raised 	Medium 	Rod	-	+	-	+	+	-	+	-	-	+	-	E. coli 	
95.		3PK1B2	Gray  	Circular 	Smooth 	Raised 	Medium 	Rod	-	+	-	+	+	-	+	-	-	+	-	E. coli 	
96.		3PM2B1	Creamy 	Circular 	Smooth 	Raised 	Medium 	Rod	-	+	-	+	+	-	+	-	-	+	-	E. coli 	
97.		3PM3B3	Creamy 	Circular 	Smooth 	Raised 	Medium 	Rod	-	+	-	+	+	-	+	-	-	+	-	E. coli 	
98.		3PS3B4	Creamy 	Circular 	Smooth 	Raised 	Medium 	Rod	-	+	-	+	+	-	+	-	-	+	-	E. coli 	
99.		3BM3B5	Creamy 	Circular 	Smooth 	Raised 	Medium 	Rod	-	+	-	-	+	+	+	+	-	+	-	Salmonella Spp	
100.		3BM1B3	Creamy 	Circular 	Smooth 	Raised 	Medium 	Rod	-	+	-	-	+	+	+	+	-	+	-	Salmonella Spp	
101.		3PS3B4	Creamy 	Circular 	Smooth 	Raised 	Medium 	Rod	-	+	-	-	+	+	+	+	-	+	-	Salmonella Spp	
102.		3BK2B1	Creamy 	Circular 	Smooth 	Raised 	Medium 	Rod	-	-	-	-	-	+	+	-	-	+	-	Shigella Spp	
103.		3OM3B4	Creamy 	Circular 	Smooth 	Raised 	Medium 	Rod	-	-	-	-	-	+	+	-	-	+	-	Shigella Spp	
104.		3PK3B1	Red 	Circular 	Smooth 	Raised 	Medium 	Rod	-	+	-	-	-	+	+	-	-	-	-	Serratia Spp	
105.		3BK2B1	Red 	Circular 	Smooth 	Raised 	Medium 	Rod	-	+	-	-	-	+	+	-	-	-	-	Serratia Spp	


Table 11.  Morphological and biochemical characterization of yeast isolates from overripe fruit samples
No	
Isolate Code	Colony Morphology on SDA	
Colony Color on BiGGY Agar	
Germ Tube 	
Presumptive  Species	
		Color	Shape 	Size	Texture 	Elevation				
1.		3BK1Y1	Creamy	Circular	Medium 	Smooth	Convex	Brown	-	Candida Spp.	
2.		3BK1Y2	Creamy	Circular	Large 	Smooth	Flat	Dark brown/yellow halo	+	Candida albican	
3.		3BK1Y3	White	Circular	Medium 	Smooth	Convex	Dark brown/yellow halo	-	Saccharomyces Spp 	
4.		3BK2Y2	Creamy	Circular	Small 	Smooth	Convex	Brown	-	Candida Spp.	
5.		3BK2Y3	White	Circular	Large 	Smooth	Convex	Dark brown/yellow halo	-	Saccharomyces Spp 	
6.		3BK2Y4	Creamy	Circular	Medium 	Smooth	Convex	Black/yellow halo	+	Candida albican	
7.		3BK3Y1	Creamy	Circular	Medium 	Smooth	Convex	Dark brown/yellow halo	-	Candida Spp.	
8.		3BK3Y4	White	Circular	Medium 	Smooth	Convex	Brown/black center	-	Saccharomyces Spp 	
9.		3BK4Y3	Creamy	Circular	Medium 	Smooth	Convex	Dark brown/yellow halo	-	Candida Spp.	
10.		3BM1Y1	Creamy	Circular	Medium 	Smooth	Convex	Dark brown/yellow halo	-	Candida Spp.	
11.		3BM1Y3	White	Circular	Large 	Smooth	Flat	Black/yellow halo		Saccharomyces Spp 	
12.		3BM2Y2	Creamy	Circular	Medium 	Smooth	Convex	Dark brown/yellow halo	-	Candida Spp.	
13.		3BM2Y3	White	Circular	Medium 	Smooth	Convex	Dark brown/yellow halo	-	Saccharomyces Spp 	
14.		3BM3Y1	Creamy	Circular	Medium 	Smooth	Convex	Brown/black center	+	Candida albican	
15.		3BM3Y2	Creamy	Circular	Small 	Smooth	Convex	Dark brown/yellow halo	-	Candida Spp.	
16.		3BM4Y3	Creamy	Circular	Medium 	Smooth	Convex	Brown/black center	-	Candida Spp.	
17.		3BM4Y3	White	Circular	Medium 	Smooth	Convex	Brown	-	Saccharomyces Spp 	
18.		3BS1Y1	Creamy	Circular	Large 	Smooth	Flat 	Dark brown/yellow halo	+	Candida albican	
19.		3BS1Y3	Creamy	Circular	Small 	Smooth	Convex	Dark brown/yellow halo	-	Candida Spp.	
20.		3BS2Y3	Creamy	Circular	Large 	Smooth	Convex	Brown/black center	-	Candida Spp.	
21.		3BS2Y3	White	Circular	Small 	Smooth	Convex	Dark brown/yellow halo	-	Saccharomyces Spp 	
22.		3BS3Y1	Creamy	Circular	Medium 	Smooth	Convex	Brown	-	Candida Spp.	
23.		3BS3Y3	White	Circular	Medium 	Smooth	Convex	No Growth	-	Saccharomyces Spp 	
24.		3BS4Y2	Creamy	Circular	Large 	Smooth	Flat	Dark brown/yellow halo	-	Candida Spp.	
25.		3BS4Y3	White	Circular	Small 	Smooth	Convex	Dark brown/yellow halo	-	Saccharomyces Spp 	
26.		3OK1Y1	Creamy	Circular	Large 	Smooth	Convex	Dark brown/yellow halo	+	Candida albican	
27.		3OK1Y2	Creamy	Circular	Medium 	Smooth	Convex	Dark brown/yellow halo	-	Candida Spp.	
28.		3OK1Y3	White	Circular	Large 	Smooth	Convex	Black/yellow halo	-	Saccharomyces Spp 	
29.		3OK2Y3	Creamy	Circular	Small 	Smooth	Convex	Dark brown/yellow halo	-	Candida Spp.	
30.		3OK3Y1	Creamy	Circular	Medium 	Smooth	Convex	Dark brown/yellow halo	-	Candida Spp.	
31.		3OK3Y4	White	Circular	Medium 	Smooth	Convex	Brown/black center	-	Saccharomyces Spp 	
32.		3OK4Y1	Creamy	Circular	Large 	Smooth	Convex	Dark brown/yellow halo	-	Candida Spp.	
33.		3OK4Y3	White	Circular	Medium 	Smooth	Convex	Black/yellow halo	-	Saccharomyces Spp 	
34.		3OM1Y1	Creamy	Circular	Large 	Smooth	Flat 	Dark brown/yellow halo	+	Candida albican	
35.		3OM1Y2	Creamy	Circular	Small 	Smooth	Convex	Brown/black center	-	Candida Spp.	
36.		3OM1Y4	White	Circular	Large 	Smooth	Convex	Brown/black center	-	Saccharomyces Spp 	
37.		3OM2Y3	Creamy	Circular	Small 	Smooth	Convex	Brown/black center	-	Candida Spp.	
38.		3OM2Y3	White	Circular	Small 	Smooth	Convex	Brown	-	Saccharomyces Spp 	
39.		3OM3Y1	Creamy	Circular	Medium 	Smooth	Convex	Brown/black center	-	Candida Spp.	
40.		3OM4Y2	Creamy	Circular	Small 	Smooth	Convex	Brown/black center	-	Candida Spp.	
41.		3OS1Y1	Creamy	Circular	Medium 	Smooth	Convex 	Dark brown/yellow halo	-	Candida Spp.	
42.		3OS1Y3	White	Circular	Medium 	Smooth	Convex	No Growth	-	Saccharomyces Spp 	
43.		3OS2Y2	Creamy	Circular	Small 	Smooth	Convex	Brown	-	Candida Spp.	
44.		3OS3Y3	Creamy	Circular	Large 	Smooth	Flat 	Black/yellow halo	-	Candida Spp.	
45.		3OS3Y4	White	Circular	Medium 	Smooth	Convex	No Growth	-	Saccharomyces Spp 	
46.		3PK1Y1	Creamy	Circular	Medium 	Smooth	Convex	Black/yellow halo	-	Candida Spp.	
47.		3PK1Y3	Creamy	Circular	Large 	Smooth	Convex	Brown	+	Candida albican	
48.		3PK1Y3	White	Circular	Medium 	Smooth	Flat	Black/yellow halo	-	Saccharomyces Spp 	
49.		3PK2Y4	Creamy	Circular	Medium 	Smooth	Convex	Black/yellow halo	+	Candida albican	
50.		3PK2Y4	White	Circular	Small 	Smooth	Convex	Brown	-	Saccharomyces Spp 	
51.		3PK4Y3	White	Circular	Small 	Smooth	Convex	Dark brown/yellow halo	-	Saccharomyces Spp 	
52.		3PM1Y1	Creamy	Circular	Large 	Smooth	Flat	Dark brown/yellow halo	-	Candida Spp.	
53.		3PM1Y2	Creamy	Circular	Large 	Smooth	Flat	Black/yellow halo	+	Candida albican	
54.		3PM1Y4	Creamy	Circular	Large 	Smooth	Flat	Dark brown/yellow halo	-	Candida Spp.	
55.		3PM1Y3	Creamy	Circular	Large 	Smooth	Flat	Dark brown/yellow halo	+	Candida albican	
56.		3PM1Y5	White	Circular	Medium 	Smooth	Convex	Brown/black center	-	Saccharomyces Spp 	
57.		3PM2Y1	Creamy	Circular	Medium 	Smooth	Convex	Brown/black center	-	Candida Spp.	
58.		3PM2Y3	White	Circular	Medium 	Smooth	Convex	Dark brown/yellow halo	-	Saccharomyces Spp 	
59.		3PM3Y2	Creamy	Circular	Medium 	Smooth	Convex	Dark brown/yellow halo	-	Candida Spp.	
60.		3PM3Y3	Creamy	Circular	Large 	Smooth	Convex	Brown	-	Candida Spp.	
61.		3PM3Y5	White	Circular	Large 	Smooth	Flat	Dark brown/yellow halo	-	Saccharomyces Spp 	
62.		3PM4Y2	Creamy	Circular	Medium 	Smooth	Convex	Dark brown/yellow halo	+	Candida albican	
63.		3PS1Y1	Creamy	Circular	Large 	Smooth	Flat 	Dark brown/yellow halo	-	Candida Spp.	
64.		3PS2Y1	Creamy	Circular	Large 	Smooth	Convex	Dark brown/yellow halo	-	Candida Spp.	
65.		3PS2Y4	White	Circular	Large 	Smooth	Flat	Dark brown/yellow halo	-	Saccharomyces Spp 	
66.		3PS3Y1	Creamy	Circular	Medium 	Smooth	Convex	Dark brown/yellow halo	+	Candida albican	
67.		3PS3Y2	Creamy	Circular	Large	Smooth	Flat	Brown/black center	-	Candida Spp.	
68.		3PS3Y4	White	Circular	Large 	Smooth	Flat	Black/yellow halo	-	Saccharomyces Spp 	
69.		3PS4Y2	Creamy	Circular	Medium 	Smooth	Convex	Dark brown/yellow halo	-	Candida Spp.	
70.		3PS4Y3	Creamy	Circular	Large 	Smooth	Flat 	Black/yellow halo	+	Candida albican	
71.		3PS4Y3	White	Circular	Medium 	Smooth	Convex	Brown	-	Saccharomyces Spp 	
Table 12.  Morphological characterization of mould isolates from overripe fruit samples grown on PDA
No	Code	Front color	Rev side  	Growth Pattern	Pre  Species	
1.		3BK1M1	Deep Green 	Creamy 	Dominant 	Aspergillus Spp.	
2.		3BK1M2	Black 	Balck 	Dominant  	Alternaria Spp.	
3.		3BK1M4	Yellow	Brown	Poor  	Fuzarium Spp.	
4.		3BK1M6	Greenish gray 	Creamy	Dominant	Penicellium Spp.	
5.		3BK2M1	Gray	Black 	Dominant 	Aspergillus Spp.	
6.		3BK2M2	Black 	Balck 	Dominant  	Alternaria Spp.	
7.		3BK2M4	Yellow	Brown	Poor  	Fuzarium Spp.	
8.		3BK2M5	White 	White 	Dominant 	Mucor Spp	
9.		3BK4M1	Black 	Creamy 	Dominant 	Aspergillus Spp.	
10.		3BM1M1	Gray	Black 	Dominant 	Aspergillus Spp.	
11.		3BM1M2	Black 	Balck 	Dominant  	Alternaria Spp.	
12.		3BM1M4	Yellow	Brown	Poor  	Fuzarium Spp.	
13.		3BM1M7	White 	Light brown 	Dominant 	Rhizopus Spp.	
14.		3BM2M1	Black 	Creamy 	Dominant 	Aspergillus Spp.	
15.		3BM2M4	Yellow	Brown	Poor  	Fuzarium Spp.	
16.		3BM2M6	Greenish gray 	Creamy	Dominant	Penicellium Spp.	
17.		3BM4M1	Deep Green 	Creamy 	Dominant 	Aspergillus Spp.	
18.		3BM4M2	Black 	Balck 	Dominant  	Alternaria Spp.	
19.		3BS1M1	Black 	Creamy 	Dominant 	Aspergillus Spp.	
20.		3BS2M1	Black 	Creamy 	Dominant 	Aspergillus Spp.	
21.		3BS2M5	White 	White 	Dominant 	Mucor Spp	
22.		3BS2M6	Greenish gray 	Creamy	Dominant	Penicellium Spp.	
23.		3BS3M1	Black 	Creamy 	Dominant 	Aspergillus Spp.	
24.		3BS3M2	Black 	Balck 	Dominant  	Alternaria Spp.	
25.		3BS3M4	Yellow	Brown	Poor  	Fuzarium Spp.	
26.		3BS3M7	White 	Light brown 	Dominant 	Rhizopus Spp.	
27.		3BS4M4	Yellow	Brown	Poor  	Fuzarium Spp.	
28.		3OK1M1	Gray	Black 	Dominant 	Aspergillus Spp.	
29.		3OK1M3	White 	Light yellow	Poor 	Botirytis Spp.	
30.		3OK1M6	Greenish gray 	Creamy	Dominant	Penicellium Spp.	
31.		3OK2M1	Gray	Black 	Dominant 	Aspergillus Spp.	
32.		3OK2M6	Greenish gray 	Creamy	Dominant	Penicellium Spp.	
33.		3OK3M4	Yellow	Brown	Poor  	Fuzarium Spp.	
34.		3OK3M6	Greenish gray 	Creamy	Dominant	Penicellium Spp.	
35.		3OK3M7	White 	Light brown 	Dominant 	Rhizopus Spp.	
36.		3OK4M2	Black 	Balck 	Dominant  	Alternaria Spp.	
37.		3OM1M1	Gray	Black 	Dominant 	Aspergillus Spp.	
38.		3OM1M4	Yellow	Brown	Poor  	Fuzarium Spp.	
39.		3OM1M6	Greenish gray 	Creamy	Dominant	Penicellium Spp.	
40.		3OM2M1	Gray	Black 	Dominant 	Aspergillus Spp.	
41.		3OM2M2	Black 	Balck 	Dominant  	Alternaria Spp.	
42.		3OM2M6	Greenish gray 	Creamy	Dominant	Penicellium Spp.	
43.		3OM3M1	Gray	Black 	Dominant 	Aspergillus Spp.	
44.		3OM3M3	White 	Light yellow	Poor 	Botirytis Spp.	
45.		3OS1M1	Gray	Black 	Dominant 	Aspergillus Spp.	
46.		3OS1M2	Black 	Balck 	Dominant  	Alternaria Spp.	
47.		3OS1M3	White 	Light yellow	Poor 	Botirytis Spp.	
48.		3OS2M1	Deep Green 	Creamy 	Dominant 	Aspergillus Spp.	
49.		3OS2M5	White 	White 	Dominant 	Mucor Spp	
50.		3OS3M6	Greenish gray 	Creamy	Dominant	Penicellium Spp.	
51.		3OS4M4	Yellow	Brown	Poor  	Fuzarium Spp.	
52.		3PK1M1	Gray	Black 	Dominant 	Aspergillus Spp.	
53.		3PK1M2	Black 	Balck 	Dominant  	Alternaria Spp.	
54.		3PK1M5	White 	White 	Dominant 	Mucor Spp	
55.		3PK2M1	Gray	Black 	Dominant 	Aspergillus Spp.	
56.		3PK2M2	Black 	Balck 	Dominant  	Alternaria Spp.	
57.		3PK2M4	Yellow	Brown	Poor  	Fuzarium Spp.	
58.		3PK2M5	White 	White 	Dominant 	Mucor Spp	
59.		3PK3M1	Black 	Creamy 	Dominant 	Aspergillus Spp.	
60.		3PK3M6	Greenish gray 	Creamy	Dominant	Penicellium Spp.	
61.		3PM1M1	Black 	Creamy 	Dominant 	Aspergillus Spp.	
62.		3PM1M6	Greenish gray 	Creamy	Dominant	Penicellium Spp.	
63.		3PM1M7	White 	Light brown 	Dominant 	Rhizopus Spp.	
64.		3PM2M1	Gray	Black 	Dominant 	Aspergillus Spp.	
65.		3PM2M2	Black 	Balck 	Dominant  	Alternaria Spp.	
66.		3PM2M6	Greenish gray 	Creamy	Dominant	Penicellium Spp.	
67.		3PM3M1	Deep Green 	Creamy 	Dominant 	Aspergillus Spp.	
68.		3PM3M4	Yellow	Brown	Poor  	Fuzarium Spp.	
69.		3PS1M1	Deep Green 	Creamy 	Dominant 	Aspergillus Spp.	
70.		3PS1M6	Greenish gray 	Creamy	Dominant	Penicellium Spp.	
71.		3PS2M1	Gray	Black 	Dominant 	Aspergillus Spp.	
72.		3PS2M2	Black 	Balck 	Dominant  	Alternaria Spp.	
73.		3PS2M4	Yellow	Brown	Poor  	Fuzarium Spp.	
74.		3PS2M5	White 	White 	Dominant 	Mucor Spp	
75.		3PS2M7	White 	Light brown 	Dominant 	Rhizopus Spp.	
76.		3PS3M3	White 	Light yellow	Poor 	Botirytis Spp.	
77.		3PS3M4	Yellow	Brown	Poor  	Fuzarium Spp.	
78.		3PS4M5	White 	White 	Dominant 	Mucor Spp	
79.		3PS4M6	Greenish gray 	Creamy	Dominant	Penicellium Spp.	
