# Supplementary material for: Evaluation of microbiome and physico-chemical profiles of fresh fruits of Musa paradisiaca, Citrus sinensis and Carica papaya at different ripening stages: Implication to quality and safety management
Source: PLoS One. 2024 Jan 30;19(1):e0297574. doi: 10.1371/journal.pone.0297574 (PMC10826968; doi:10.1371/journal.pone.0297574)
Supplement: S1 Table — (RTF) [file pone.0297574.s003.rtf]

Table 1. MALDI-TOF MS identified bacterial species 
Bacterial Species 	Isolate Code	Score value	Source Sample 	
Alcaligenes faecalis	JUB47	2.22	Banana S1	
Alcaligenes faecalis	JUB46	2.17	Banana S2	
Alcaligenes faecalis	JUB59	2.27	Banana S2	
Alcaligenes faecalis	JUB5	1.75	Banana S3	
Bacillus cereus	JUB1	2.09	Banana S1	
Bacillus cereus	JUB2	2.11	Banana S1	
Bacillus cereus	JUB3	1.72	Banana S2	
Bacillus cereus	JUB17	2.14	Banana S3	
Bacillus cereus	JUB18	1.89	Banana S3	
Bacillus cereus	JUB19	2.10	Banana S2	
Bacillus cereus	JUB32	2.26	Banana S3	
Bacillus cereus	JUB33	2.30	Banana S2	
Pseudomonas putida	JUB34	2.21	Banana S2	
Alcaligenes faecalis	JUO7	2.12	Orange S1	
Alcaligenes faecalis	JUO8	1.47	Orange S1	
Alcaligenes faecalis	JUO38	2.30	Orange S2	
Alcaligenes faecalis	JUO39	2.21	Orange S2	
Alcaligenes faecalis	JUO50	2.15	Orange S3	
Alcaligenes faecalis	JUO51	1.89	Orange S3	
Bacillus cereus	JUO9	2.10	Orange S2	
Bacillus cereus	JUO24	2.08	Orange S3	
Morganella morganii	JUO60	2.40	Orange S2	
Staphylococcus sciuri	JUO10	2.21	Orange S2	
Staphylococcus epidermidis	JUO25	2.12	Orange S3	
Alcaligenes faecalis	JUP11	2.12	Papaya S1	
Alcaligenes faecalis	JUP15	1.84	Papaya S2	
Alcaligenes faecalis	JUP64	2.37	Papaya S2	
Alcaligenes faecalis	JUP26	2.13	Papaya S3	
Bacillus cereus	JUP12	2.19	Papaya S1	
Bacillus cereus	JUP54	2.29	Papaya S2	
Bacillus cereus	JUP14	1.79	Papaya S2	
Bacillus cereus	JUP27	1.81	Papaya S3	
Bacillus cereus	JUP28	2.13	Papaya S3	
Morganella morganii	JUP52	2.31	Papaya S2	
Pseudomonas putida	JUP41	2.02	Papaya S1	
Pseudomonas putida	JUP42	1.82	Papaya S2	
Where, S1 = Mature green, S2 = Moderately ripe, S3 = Overripe stage. 
