# Supplementary material for: Evaluation of microbiome and physico-chemical profiles of fresh fruits of Musa paradisiaca, Citrus sinensis and Carica papaya at different ripening stages: Implication to quality and safety management
Source: PLoS One. 2024 Jan 30;19(1):e0297574. doi: 10.1371/journal.pone.0297574 (PMC10826968; doi:10.1371/journal.pone.0297574)
Supplement: S2 Table — (RTF) [file pone.0297574.s004.rtf]

Table 1. Physico-chemical properties of fruit samples at three different ripening stages
Sample 	pH Value	EC	Moisture content	Total Solid	Titrable acidity	Ascorbic acid	Reducing Sugar	Total Sugar	
Unripe banana	5.70	78.00	71.30	28.70	0.49	6.25	0.00	0.01	
	5.90	67.00	72.40	27.60	0.42	6.40	0.00	0.00	
	5.60	64.00	70.50	29.50	0.47	6.30	0.00	0.01	
Ripened banana	4.70	42.00	74.40	25.60	0.90	8.20	12.50	15.60	
	4.60	40.00	74.70	25.30	0.93	7.85	12.20	15.20	
	4.80	36.00	73.90	26.10	0.89	8.25	11.70	14.80	
Overripe banana	4.50	36.00	76.20	23.20	0.98	6.45	14.60	18.20	
	4.40	33.00	75.50	24.50	1.12	6.35	14.20	17.80	
	4.50	24.00	75.60	24.40	0.99	6.40	13.80	17.60	
Unripe Orange	3.50	168.00	86.80	13.20	1.25	38.80	4.05	6.80	
	3.50	157.00	85.60	14.40	1.32	39.90	4.15	7.20	
	3.60	152.00	86.20	13.80	1.26	40.60	4.02	6.40	
Ripen Orange	3.60	159.00	88.40	11.60	0.92	49.50	5.70	10.65	
	3.70	148.00	89.10	10.90	0.94	50.20	5.80	9.86	
	3.60	134.00	88.70	11.30	0.90	48.80	6.20	11.44	
Overripe Orange	4.10	143.00	85.80	14.20	0.77	40.40	8.80	12.60	
	4.20	133.00	86.20	13.80	0.72	39.60	8.60	12.20	
	3.80	122.00	84.60	15.40	0.76	42.30	9.20	13.40	
Unripe Papaya	6.02	16.80	86.20	13.80	0.05	61.60	5.20	5.65	
	6.15	14.40	87.50	12.50	0.04	62.50	4.65	5.25	
	6.08	12.30	88.10	17.90	0.04	60.80	4.04	4.76	
Ripen Papaya	5.65	5.90	92.40	7.60	0.10	68.20	6.05	7.05	
	5.72	6.10	90.60	9.40	0.12	67.80	6.10	6.80	
	5.78	5.10	89.20	10.80	0.11	68.40	6.05	7.20	
Overripe Papaya	5.67	4.00	92.70	7.30	0.14	70.20	6.25	7.88	
	5.70	4.75	91.30	8.70	0.16	69.80	6.30	7.86	
	5.63	3.20	93.10	6.90	0.15	69.60	6.35	7.90	
