# Supplementary material for: Evaluation of microbiome and physico-chemical profiles of fresh fruits of Musa paradisiaca, Citrus sinensis and Carica papaya at different ripening stages: Implication to quality and safety management
Source: PLoS One. 2024 Jan 30;19(1):e0297574. doi: 10.1371/journal.pone.0297574 (PMC10826968; doi:10.1371/journal.pone.0297574)
Supplement: S1 Fig — (RTF) [file pone.0297574.s005.rtf]

Fig. 1. Map of the study area (Shapefiles from https://data.humdata.org/dataset/cod-ab-eth)
