# Supplementary material for: Evaluation of microbiome and physico-chemical profiles of fresh fruits of Musa paradisiaca, Citrus sinensis and Carica papaya at different ripening stages: Implication to quality and safety management
Source: PLoS One. 2024 Jan 30;19(1):e0297574. doi: 10.1371/journal.pone.0297574 (PMC10826968; doi:10.1371/journal.pone.0297574)
Supplement: S3 Fig — (RTF) [file pone.0297574.s007.rtf]

S7 Figure. Peel color and maturity level of the fruits samples at different ripening stages. 
