# Supplementary material for: Evaluation of microbiome and physico-chemical profiles of fresh fruits of Musa paradisiaca, Citrus sinensis and Carica papaya at different ripening stages: Implication to quality and safety management
Source: PLoS One. 2024 Jan 30;19(1):e0297574. doi: 10.1371/journal.pone.0297574 (PMC10826968; doi:10.1371/journal.pone.0297574)
Supplement: Attachment — Submitted filename: Dawit etal 2023 to PLOS ONE.pdf [file pone.0297574.s008.pdf]

Evaluation of Microbiome and Physico-chemical Profiles of Fresh Fruits at Different Ripening Stages: Implication to Quality and Safety Management

Commented [Rev1]: Of what ?? mentioned fruit name in title

Dawit Kifle[1, 2*], Reda Nemo[3] Lata Lachisa[1] and Ketema Bacha[1]

[1] Jimma University, College of Natural Sciences, Department of Biology, Jimma, Ethiopia,

[2] Mizan-Teppi University, College of Natural and Computational Sciences, Department of Biology, Teppi, Ethiopia;

[3] Dembi Dollo University, College of Natural and Computational Sciences, Department of Biology, Dembi Dollo, Ethiopia.

[*] dawitk.galo@gmail.com

Abstract

Background: The ripening of fleshy fruits is a developmental process that involves changes in color, texture, aroma, nutrients, and diversity of microbiomes. Some microorganisms, specially, bacteria and molds are responsible for postharvest spoilage of fruits. Thus, this study is aimed at evaluating the alterations in microbiome and physico-chemical properties of selected fruits at different ripening stages. **Methods:** Totally, 108 fresh fruit samples of three ripening stages were collected and processed in this study. The biochemical methods and MALD-TOF MS were used in identification. The physico-chemical property of all samples were analyzed using standard methods. **Results:** The minimum counts (6.74± 0.48-6.76± 0.42 log CFU/mL) and the maximum count (7.51± 0.43-7.96± 0.34 log CFU/mL) of AMB in all fruit samples was observed at mature green and overripe stages of the fruits, respectively. The ripening stage has significantly affected the microbial counts ($p<0.05$) in all fruits, except counts of Enterobacteriaceae in banana and orange, and fungal counts in orange. The bacterial community of all fruits was predominated by *B. cereus* (33.7%), *A. faecalis* (17.3%), *P. putida* (15.2%), *M. morganii* (11.1%), S. sciuri (6.6%) and *S. epidermidis* (4.9%); while the fungal microbiome was constituted by *Candida spp* (33.9%) followed by *Saccharomyces* spp (18.1%) and *Aspergillus spp* (16.3%). The ripening stages has also significantly affected the physico-chemical property in all samples. Accordingly, the lowest pH (3.53) and highest content of ascorbic acid (69.87 mg/100g) were observed in mature green oranges and overripe papaya, respectively, while the maximum concentration of total sugar (17.87%) and reducing sugar (14.20%) were recorded in overripe bananas. **Conclusion:** The presence of some potential human pathogens and spoilage microorganisms in fruit samples could contribute to post-harvest product losses besides the potential health risk associated with consumption of the tainted fruits. Hence, proper safety management practices and preservation mechanisms should be developed and put in place to ensure consumers safety against pathogens besides minimizing product losses through microbial spoilage.

Key terms: Fruits, microbiomes, Physico-chemical properties, Ripening stages.

Commented [Rev2]: Which type of fruits similar in heading

Commented [Rev3]: Should not italic follow similar trend in the MS

Background

Commented [Rev4]: Introduction is very weak, please write some earlier work done in this topic and how your work is novel in this regard.

True fruits are specialized plant organs found solely in angiosperms, and these unique organs are believed to have evolved to improve seed protection and dispersal by creating attractive flesh and aroma for seed-dispersing animals [1]. They are important for human nutrition, providing energy, carbohydrates, essential amino acids, minerals, fibers, vitamins and antioxidants essential for human nutrition and health [2].

The issue of food losses is of high importance in the efforts to combat hunger, raise income and improve food security in the world's poorest countries [3]. The world is producing annually 675 million tons of fruits to meet the nutrition requirement of its population as per the statistics available in 2017 [4]. Yet, huge post-harvest losses reach in the range of 15-20% in the USA and Canada, 25% in Europe, and 30-35% in Asia and an estimated one in eight people suffer from chronic under-nourishment [5]. This depicts the extent of post-harvest losses due to the perishability and deterioration of fruits before reaching the consumer, which occurs mostly after ripening of fruits [6].

The ripening of fleshy fruits is a developmental process that involves dramatic changes in color, texture (softening of the fruit tissues), flavor, aroma, and nutrients [7]. Fleshy fruit is categorized as climacteric (including banana, papaya, apple, and tomato) and non-climacteric (including Citrus, grapes, and melon) based on differences in their respiration and ethylene biosynthesis during ripening [1]. The ethylene biosynthesis in fruits can also be affected by the metabolic activities of plant microbiomes [8].

Plant microbiomes consist of complex communities of potentially mutualistic, commensal, and pathogenic microbes colonizing the same niches in plants, including bacteria (such as *Bacillus* spp, *Staphylococcus* spp, *Pseudomonas* spp and gram-negative bacilli, and yeasts and molds) that have diverse aspects of impacts on plant growth, health, and evolution [9, 10]. The microbes that constitute the fruit microbiome can colonize the fruit either vertically, as in the case of endophytes that live within plant tissues, or horizontally from the rhizosphere, or through mixed colonization [11, 12].

The fruit microbiota might be especially important during crop storage by preventing or favoring rots, or quality loss due to sprouting, water loss, or else spoilage. Yet, pathogen-induced decay is certainly the most obvious reason for postharvest crop loss mainly caused by bacteria and molds [11]. Other study indicated that *Enterobacteriaceae* and *Staphylococcus* spp were representing the bacterial community of fresh fruits in Arba Minch [13]. Weldezgina & Muleta, (2016) also revealed that fruits and vegetable samples from Jimma town were dominated by *Bacillus* spp (32.7%) and *Enterobacteriaceae* (25%). The presence of pathogens and spoilage microorganisms can reduce fruit production.

Ethiopia's fruit production and export play a significant role in the local economy as a means of earning livelihoods for ~5 million farmers, and for generating foreign exchange revenues [5]. More than 120 million hectares of land are under fruit crops in Ethiopia, with ~55.11% of the area allocated to banana [15]. In 2018, ~8.4 million quintals of fruits were produced in the country; while bananas, mangoes, papayas, and oranges took up 60.11%, 16.02%, 7.10%, and 4.94% of the fruit production, respectively [4]. As stated in Tesfaye, (2019), the production of fruits in Ethiopia is related to climate, availability of nutrients in the soil, and the physico-chemical characteristics of the soil. Among Ethiopian regions, southwestern is well known for producing large quantities of fruits.

Southwestern Ethiopia, particularly the Bench-Sheko Zone of the Southwest Regional state is among the evergreen, naturally rich parts of the Ethiopia providing surplus production of fruits and vegetables. Banana, orange, papaya, mango and avocado are among the major fruit crops produced throughout the year 2018 [16]. However, the production of these fruits has been declining from time to time due to microbial spoilage and factors such as higher relative humidity, poor pre-and post-harvest handling, transportation and storage. So, a large ~~amount~~number of fruits has been lost because of postharvest spoilage. Thus, this study aims to assess the changes in microbiomes and physico-chemical properties of selected fruits at different ripening stages. The findings could be helpful for awareness development among those working directly or indirectly on fruit production, processing and consumption in order to ensure consumers safety and minimize product losses.

Commented [Rev5]: Please write clear objectives

Materials and Methods

Description of the Study Site

The study sites, Gura Ferda, Sheko, and Mizan-Aman Town and its surrounding districts, are located in the Bench-Sheko Zone, currently administered under the newly established Southwestern National Regional State of Ethiopia (Figure 1). The zone is located at 6^0 58' N and 35^0 45'E, with altitudinal ranges between 900 and 1810m (masl). The mean minimum and maximum annual temperature of the area are 15.23^o C and 30.17^o C, respectively with the mean annual rainfall of the area being 1850.55 mm/year. The area is largely covered with tropical deciduous forest [17].

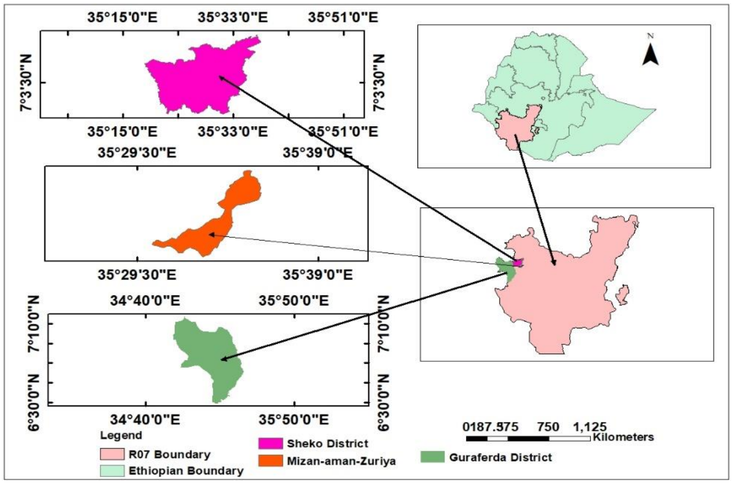

Figure 1: Map of the study area

Commented [Rev6]: Please show the surveyed places in the Map

The three study sites were selected purposively; as the area is one of the well-known sites in Ethiopia for its high fruit production throughout the year [16].

Study Design

An experimental-based cross-sectional study design was employed. Samples were taken in three phases to obtain fresh fruit samples with different ripening stages.

Sample Size and Sampling Techniques

Totally, 108 fresh fruit samples, 36 banana (*Musa paradisiaca*), 36 orange (*Citrus sinensis*) and 36 papaya (*Carica papaya*) were collected for this study. In the first phase of sample collection, 36 mature green fruit samples, 12 from each of the three fruit types, were collected and processed for both microbiological and physico-chemical analyses. This task was repeated for moderately ripe stage and overripe stage of the fruits. These stages were selected due to the assumption that fruits at these stages may exhibit significantly different Physico-chemical properties as well as changes in microbiome profile. The samples were collected separately and aseptically, put into sterile labeled polythene bags and transported using an icebox to Jimma University Research and Post-Graduate Laboratory for analysis. The samples were temporarily stored under refrigeration (4°C) until processed.

Commented [Rev7]: Author name for each fruit

Sample Preparation for Microbiological Analysis

An average of 1Kg of papaya, 600g of each banana, and orange fruit sample was weighed separately and aseptically chopped with its peel into small pieces using sterile blade and blended using an electronic blender (RL – Y66, Italy) until completely homogenized. Then, 25 g of each fruit juice was mixed in 225 mL of sterile peptone water and homogenized for 10 minutes using a shaker (Compact Shaker, D-72379 Hechingen, Germany). Then, serial dilution of each suspension was made up to 10^{-7}. Finally, 0.1 ml aliquot of each dilution was spread-plated on a sterile pre-solidified agar plates and incubated at the appropriate temperature and time. Microbial enumerations were made in triplicates following standard methods [18].

Microbial Enumeration

The viable colonies of total aerobic mesophilic bacteria (AMB), total aerobic spore-forming bacteria (ASFB), lactic acid bacteria (LAB), *Enterobacteriaceae*, Staphylococci, yeasts and molds and were enumerated in accordance with standard procedures [18, 19]. Briefly, 0.1 ml aliquot of each sample was spread plated in duplicates on Plate Count Agar (PCA) (Oxoid), De Man Rogosa Sharpe (MRS) (Microgen), Violet Red Bile Glucose Agar (VRGBA) (Microgen), Mannitol Salt Agar (MSA) (Microgen), Potato Dextrose Agar (PDA) (Microgen) for the count of AMB, LAB, *Enterobacteriaceae*, Staphylococci, and molds/yeasts, respectively. For the

enumeration of ASFB, a suspension of 10 mL was heated in a water bath at 80°C for 10 minutes to remove vegetative cells followed by inoculation of 0.1ml aliquot onto PCA and incubated at 30-32°C for 72hrs. Only plates containing a countable number of colonies (30-300 for bacteria and 10-150 for fungal colonies) were enumerated and the mean counts of the colonies were expressed in CFU ml^{-1} which later converted to Log CFU ml^{-1}.

Isolation and Characterization of Bacterial Colonies

Commented [Rev8]: Please write details about characterization, why did you not do molecular characterization of the microbes

The microbial communities (both epiphytes and endophytes) from all the fruit samples were counted, isolated, and characterized using standard methods [18, 20]. After the enumeration of AMB from Plate Count Agar (PCA), 5-10 colonies of bacteria with distinct morphological differences such as color, size, and shape were randomly picked from countable plates and aseptically transferred into test tubes containing 5 ml Nutrient Broth (Microgen) and incubated at 32° C for 24 hours. Then, a loop-full of the broth growth was streak-plated on nutrient agar and incubated at 32°C for 24 hours. Sub-culturing was done until pure colonies were obtained, and the pure culture was preserved at 4° C as slants for further characterization. Pure isolates were characterized for colony morphology (size, color, margin, and elevation), cellular morphology (shape, motility, and endospore), and further subjected to biochemical tests (KOH, catalase, Citrate, Indole, H_2S, oxidase, triple sugar and Coagulase tests) in accordance with standard methods [16]. Finally, the identified isolates were preserved in 20% glycerol at -21° C.

Identification of Dominant Isolates using MALDI-TOF MS

Representatives of suspected bacterial colonies formerly identified by the conventional biochemical method from all fruit samples were also analyzed using MALDI-TOF MS. The MALDI-TOF MS spectrometer (EXS3000 MALDI-TOF MS, China) was used to obtaining the mass spectra. The formic acid extraction procedure was achieved based on the manufacturer's guidelines for the identification of the bacterial isolates. Briefly, exact 300µl ultra-pure water was pipetted into 1.5mL centrifuge tube, 5-10mg of fresh 24h bacterial colony was picked using pipette tip and mixed fully. Then, 900µl absolute ethanol (100%) was added into the tube and well mixed by vortex mixer for 20s, centrifuged at 12000 rpm for 3min, the supernatant poured out, centrifuged again for 1min and the supernatant pipetted out carefully. After 5 minutes of drying the sample completely, 20µl lysate I (70% formic acid) was added into the tube and tip

mixed. The gram-positive bacteria were left for 5 minutes for enough lysate reaction. The, 20ul of lysate II (Acetonitrile, CAN) was added, mixed by Vortex mixer for 10s and centrifuged at 12000 rpm for 2 min. Finally, 1µl of the supernatant was pipetted onto target plate point, dried fully, after which 1µl of matrix solution was added to cover the sample, and died fully to test.

Similar to Zhang et al., (2022), the spectra were produced by applying the Compass Satellite software, and the Microflex LT device was used for fast and accurate identification of bacterial colonies isolated from the fruit samples. A spectra score of 2.300–3.000 indicates that the identification of bacterial species has high reliability, 2.000–2.299 indicates the identification of possible species, 1.700–1.999 indicates the identification of possible genus, and 0.000–1.699 indicates that the identification result is unreliable. *E. coli* ATCC 8739 was used as the standard strain before the detection of each test sample.

Commented [Rev9]: What about replication, did you repeated the experiments

Isolation and characterization of mould and yeast colonies

After enumeration on PDA plates, 5-7 yeast and mould colonies of different morphology, were aseptically transferred to separate 5 mL of PDB tubes, and PDA plates, respectively. A pure culture of each colony on each medium was obtained by repeated sub-culturing. The pure cultures of molds were aseptically transferred onto PDA slants, and incubated at room temperature again for 3 days, and stored at 4° C for further use. The pure cultures of yeasts were aseptically transferred onto PDB and incubated at 30° C for 48 hours after which stored at 4°C for further identification [19]. Then, the pure isolates of molds and yeasts were identified by microscopic observation of hyphal structure (molds), and sporangiospore and cellular structure by staining using Lactophenol cotton blue, using a taxonomic key as a morphological reference [22]. The *Candida Spp* were then screened using BiGGY Agar and germ tube test [23]. After identification, the isolates were preserved in 20% Glycerol at -20 ^{0}C.

Commented [Rev10]: Simmilr comments why not molecular characterization, how many replication, did you repeated experiments ?

Formatted: Highlight

Determination of physico-chemical properties of fruit samples

Sample preparation

The samples collected directly from the local farms of the Bench-Sheko residents were transported to Jimma University Research and PG Lab. Each sample was separately and

aseptically soaked into sterile water and washed. Then, the fruit samples were separately chopped into small pieces (with peel) and blended using an electronic blender (RL-Y66, Italy) so as to make composite juice of each fruit type. After vigorously shaken for 10 minutes using a shaker (Compact Shaker, D-72379, Germany), the juices were stored at 4ºC for further Physico-chemical tests [24].

Peel color

Banana peel color was considered for classification using the standard ripeness classification of bananas as stated in Mazen & Nashat, (2019). This scale categorizes bananas into seven stages of ripening: 1 = mature green; 2 = light green; 3 = half yellow–half green; 4 = three-quarters yellow with green; 5 = yellow with green tips; 6 = full yellow; and 7 = yellow with brown spots. For this study, three stages of ripeness were considered: stage 1 (mature green), stage 5 (yellow with green tips), and stage7 (yellow with brown spots) were considered for the collection of banana samples. For orange and papaya fruit samples, mature green (fully green), moderately ripe (yellow with a trace of green), and over-ripe (fully deep yellow) were considered [24, 26].

Determination of pH

To determine the pH content of the fruit samples, exactly 10g of each fruit sample was placed in a 50mL beaker containing 12mL distilled water, and blended using an electronic homogenizer (RL–Y66, Italy) until smooth. Then, pH was measured using a digital pH meter (pH–013, China), pipetting 10ml of the homogenized sample into a beaker [27].

Moisture and total solid content

To determine the moisture content, clean oven-dried beakers of 50 ml capacity was weighed and recorded (W1). Then, 10gm of each sample was separately homogenized, weighed, and added into pre-weighed dried beaker (W2) and oven-dried at 105 °C for 2 h. After 2 h of drying, the sample was relocated to a desiccator, and weight (W3) was recorded as in AOAC Official Method 934.06 [28]. Percentage of water content was determined as a ratio of differences between W2 and W3 to W2 and W1 multiplied by 100 as given below. Furthermore, total solid content was determined by subtracting the % moisture content from the total (100%).

$$\%\text{ of Moisture Content } = \frac{W2 - W3}{W2 - W1} X\ 100$$

Total Solid Content = 100 – Moisture content

Titrable acidity (TA)

The TA of fruits samples was determined by separately homogenizing 5 gm of each sample in 50 ml distilled water and filtered through Whatman No.1 filter paper. Then, about 9ml of the homogenate was pipetted into a beaker into which 3 drops of 1 % phenolphthalein indicator were added titrated with 0.1 N NaOH solutions until pink color was observed. The TA was calculated as percent acid as described earlier in AOAC-Official-Method-942.15 [29] using the formula given below. Finally, the TA is calculated as % of mallic acid for banana and % of citric acid for orange and papaya as follows:

$$\%\text{ of Titrable acidity} = \frac{N\ NaOH\ X\ VNaOH\ X\ F\ acid}{Volume\ of\ Jucie\ used} X\ 100$$

Where: N_{NaOH} is the normality of NaOH used (g L^{-1}), V_{NaOH} is the volume of NaOH solution consumed (L), F_{acid} is a factor equivalent weight of the acid in the fruit juice sample equal to 0.067 for citric acid and 0.064 for mallic acid.

Ascorbic acid content

The ascorbic acid content in each fruit sample was determined by titration using 0.1 M iodine solution. For each sample, 25 mL of blended juice was poured into a 250 mL volumetric flask, and 10 drops of a pre-prepared 1% soluble starch solution was added. The mixed solution was titrated with 0.1 M of iodine solution until a blue color was observed to persist for 15 s. Before determination of ascorbic acid content of the fruit samples, the titration method was optimized using standard solutions of ascorbic acid, which was titrated with 0.1 M iodine solution. Each measurement was performed in triplicates, and quantified in mg per 100 g of fruit juice [30].

$$\text{Ascorbic Acid (mg/100g)} = \frac{\text{VI X FAA}}{\text{Mass of the juice sample used}} \text{X 100}$$

Where: V_I = the volume of iodine solution consumed (L), F_{AA} = a conversion factor of the ascorbic acid consumed with iodine solution (molarity of iodine times the molecular mass of ascorbic acid).

Determination of reducing sugars and total sugars

The reducing and total sugar contents of all samples was determined using Lane and Eynon titration method, in accordance with AOAC Official Method 923.09 [31] and FSSAI, (2016). Accordingly, to determine the Fehling factor, 4.75g of pure sucrose was weighed and transferred to 500 ml volumetric flask with 50 ml distilled water. Then, exactly 5 ml of conc. HCl was added and allowed standing for 24 hrs. The solution neutralized with 0.1N NaOH using phenolphthalein as end point indicator and made up to volume with distilled water. Then, the reaction was mixed well and 25 ml of the solution was transferred to a 100 ml volumetric flask and made up to volume with distilled water (1 ml = 2.5 mg of invert sugar). Then, the solution was transferred to a burette having an offset tip and titrated against Fehling's solution until the blue color disappears to a brick-red, and the titer was noted as V_1. Therefore,

$$\text{Fehling Factor (g of Invert Sugar)} = \frac{\text{V1 X 2.5}}{1000} = \text{V1 X 0.0025}$$

Where: W = Weight of the sample

V_1 = Volume of sucrose solution (titer) required for complete Fehling's reduction

Secondly, to determine the reducing sugar content, 10g of each sample was accurately weighed and transferred to 500 ml volumetric flask containing 100 ml distilled water and neutralized with 0.1N NaOH solution to phenolphthalein end point. Then 10 ml of neutral lead acetate solution was added, mixed well and let stand for 10 min. Then, potassium oxalate solution was added drop by drop until there is no further precipitation. The solution was mixed well and made up to volume with distilled water, and filtered through Whatman No. 1 filter paper. The filtrate was finally transferred to a 50 ml burette having an off-set tip. Then, to conduct the preliminary titration, 5 ml each of Fehling A and B solutions was pipette into 250 ml conical flask, mixed in 10 ml water and a few glass beads was added. Then, the sugar solution from the burette was added drop wise and the solution was heated to boiling, and 3 drops of methylene blue added as an indicator. The addition of the sugar solution drop wise was continued until the blue color

disappears to a brick-red endpoint within boiling period of 3 minutes, and the titer value was noted down as V_2. For final titration, 5 ml each of Fehling A and B solutions was pipetted into a 250 ml conical flask and sample solution about 1.0 ml less than titer value in the preliminary titration (V_2) was added. The flask was heated to boiling, and titration proceeds as above. The titer value was noted down as V_3. The titration was performed in duplicate and the average was recorded. Based on the factor for Fehling's solution, V_3 ml sample solution contains 0.0025 V_1 g reducing sugar (as invert sugar). Therefore,

$$\text{Reducing sugar in the sample (\%)} = \frac{0.0025 \text{ x V1 x V2}}{\text{V3 x W}} X100$$

Where: W = Weight of the sample

V_1 = Volume of sucrose solution (titer) required for complete Fehling's reduction

V_2 = Dilution volume for the sample (V_1 – 1 ml)

V_3 = Volume of clarified sample solution (titer) required for Fehling's reaction

Thirdly, to determination of total reducing sugars, an aliquot of 50 ml of the clarified, sample filtrate was pipetted into a 100 ml volumetric flask, 5 ml of conc. HCl was added and allowed standing at room temperature for 24 hours. Then, neutralized with 0.1N NaOH solution using phenolphthalein as indicator, and made up to volume with distilled water and transferred to 50ml burette having an offset tip. Then, the titration was performed as in reducing sugars, and the titer noted as V_4. The total reducing sugars in V_4 could be calculated as V_4 ml = 0.0025 × V_1 g [32]. However, as 50 ml of the sample solution is diluted twice (50 ml to 100 ml) after hydrolysis, dilution volume of the sample is $2V_2$. Therefore,

$$\text{Total Reducing sugar in the sample (\%)} = \frac{0.0025 \text{ x V1 x 2V2}}{\text{V4 x W}} X100$$

Finally, the amount of sucrose and total sugar in the sample was calculated as:

$$\text{Sucrose in the sample (\%)} = (\text{\% Total reducing sugars} - \text{\% Reducing sugars}) \times 0.95$$

$$\text{Total sugars in the sample (\%)} = (\text{\% Reducing sugars} + \text{\% Sucrose})$$

Data analysis

The obtained data were statistically analyzed using the SPSS version 20 software package. The differences among the triplicate data obtained from microbial counts and the physico-chemical analysis were tested using one-way ANOVA, Duncan's multiple range tests with $p < 0.05$ being considered as significance.

Commented [Rev11]: Did you repeated experiments? How did you handle data from repeated experiments? Did you do homogeneity test? What about normality test ? in between it is not clear you used raw data or transformed data in analysis

Results

Microbial enumeration in fruit samples at different ripening stages

Commented [Rev12]: All the results must be supported with statistical value

The microbial load of the fruits at different stages of maturity: mature green, ripe and overripe showed a significant difference. Accordingly, the maximum AMB was observed in overripe banana (7.96 ± 0.34 Log $CFUg^{-1}$) followed by over-ripe papaya (7.82 ± 0.32 Log $CFUg^{-1}$) while the minimum count was observed in mature green orange (6.74 ± 0.48 Log $CFUg^{-1}$). Relatively lower counts were recorded for Enterobacteriaceae (1.78 ± 1.57 - 3.34 ± 0.28 Log $CFUg^{-1}$) and Staphylococci (2.06 ± 1.53 to 3.73 ± 0.29 Log $CFUg^{-1}$) in the three fruits (Table 1). Overall, the microbial load increased with progress in the fruit's maturity stages from mature green through ripe to overripe with a significant difference ($P<0.05$) between the stages. However, there is no significant difference ($P> 0.05$) in counts of Enterobacteriaceae in bananas, and yeast and molds in orange at different ripening stages (Table 1).

Table1. Mean microbial counts (Log CFU ml^{-1} ± SD) in fruit samples at different ripening stages, Bench-Sheko Zone, 2021

	**Banana *(Musa paradisiaca)***			**Orange *(Citrus sinensis)***			**Papaya *(Carica papaya)***		
**Microbe**	**S1**	**S2**	**S3**	**S1**	**S2**	**S3**	**S1**	**S2**	**S3**
AMB	6.76± 0.42[c]	7.60 ± 0.42[b]	7.96 ± 0.34[a]	6.74 ± 0.48 [c]	6.81 ± 0.45 [b]	7.51 ± 0.43 [a]	6.79 ± 0.47 [c]	7.25 ± 0.33 [b]	7.82 ± 0.32 [a]
ASFB	4.77 ± 0.39[c]	6.07 ± 0.38[b]	6.87 ± 0.29[a]	4.26± 0.51 [c]	5.41 ± 0.35 [b]	6.07 ± 0.11 [a]	4.63 ± 0.47 [c]	5.52 ± 0.42 [b]	6.24 ± 0.33 [a]
LAB	4.77 ± 0.71[c]	5.61 ± 0.59[b]	6.10 ± 0.39[a]	4.95 ± 0.25 [b]	5.33 ± 0.53 [a]	5.68 ± 0.42 [a]	5.06 ± 0.27 [c]	5.76 ± 0.39 [b]	6.14 ± 0.15 [a]
Entero	2.32 ± 1.73[a]	2.08 ± 1.54[a]	2.93 ± 1.53[a]	1.87 ± 1.65 [a]	1.78 ± 1.57 [a]	2.09 ± 1.55 [a]	2.75 ± 1.29 [ab]	1.86 ± 1.65[b]	3.34 ± 0.28 [a]
Staph	2.08 ± 1.55[b]	3.25 ± 0.38[a]	3.37 ± 0.29[a]	2.17 ± 1.62 [b]	2.21 ± 1.66 [b]	3.49 ± 0.36 [a]	2.06 ± 1.53 [b]	3.02 ± 1.02 [a]	3.73 ± 0.29 [a]
Yeasts & moulds	3.87 ± 0.43[c]	4.38 ± 0.47[b]	4.97 ± 0.24[a]	3.74 ± 0.48 [a]	3.92 ± 0.39 [a]	4.03 ± 0.43 [a]	4.09 ± 0.23 [b]	4.21 ± 0.37 [b]	4.79 ± 0.41 [a]

Where, AMB – total aerobic mesophilic bacteria, ASFB - total aerobic spore-forming bacteria, LAB - lactic acid bacteria, *Entero* – *Enterobacteriaceae*, Staph – *Staphylococcus* spp, S1 – Mature green, S2 – Moderately ripe, S3 – Overripe stage. Different letters along figures in a column indicate a significant difference ($p < 0.05$); the same letters indicate no significant difference ($p > 0.05$).

Biochemical and MALD-TOF MS identification of bacterial isolates

In this study over-all, 243 bacterial isolates were identified using the conventional biochemical methods, and 36 representative isolates by MALD-TOF MS technique. Among nine categories of morphologically different bacterial colonies isolated in this study, seven of them were identified at genera level using the biochemical methods. These include *Bacillus spp, Pseudomonas spp, Staphylococcus spp, E. coli, Salmonella spp, Shigella spp and Serratia Species*. Two of them remained unidentified by the conventional method, and later identified as *Alcaligenes faecalis* and *Morganella morganii,* using MALD-TOF MS technique.

In the present study, 36 bacterial isolates from top-five most dominant strains were randomly selected as representative, among which 97.2% of them were identified to species/genera level using MALDI-TOF MS. Then, various species of the isolates were recognized by matching their spectra with spectral profiles stored in the MALDI Biotyper database. Accordingly, 27 (75%) of the bacterial isolates were correctly identified at species level, by MALDI-TOF MS fingerprinting with a score value ≥2.00, 8 (22.2%) isolates were identified at genera level with a score value between 1.70-1.99, and only one isolate (2.8%) assumed to be *Alcaligenes spp* has shown a score value <1.700. In this study, 6 species of bacteria were identified by the MALD-TOF MS, including *B. cereus*, *A. faecalis*, *P. putida, M. morganii, S. sciuri and S. epidermidis* (Table 2).

Table 2. Bacterial Species Identified using MALDI-TOF MS

Commented [Rev13]: Why not molecular analysis ??

Bacterial Species	Isolate Code	Score value	Source Sample
Alcaligenes faecalis	JUB47	2.22	Banana S1
Alcaligenes faecalis	JUB46	2.17	Banana S2
Alcaligenes faecalis	JUB59	2.27	Banana S2
Alcaligenes faecalis	JUB5	1.75	Banana S3
Bacillus cereus	JUB1	2.09	Banana S1
Bacillus cereus	JUB2	2.11	Banana S1
Bacillus cereus	JUB3	1.72	Banana S2
Bacillus cereus	JUB17	2.14	Banana S3
Bacillus cereus	JUB18	1.89	Banana S3
Bacillus cereus	JUB19	2.10	Banana S2
Bacillus cereus	JUB32	2.26	Banana S3
Bacillus cereus	JUB33	2.30	Banana S2
Pseudomonas putida	JUB34	2.21	Banana S2
Alcaligenes faecalis	JUO7	2.12	Orange S1
Alcaligenes faecalis	JUO8	1.47	Orange S1
Alcaligenes faecalis	JUO38	2.30	Orange S2
Alcaligenes faecalis	JUO39	2.21	Orange S2
Alcaligenes faecalis	JUO50	2.15	Orange S3
Alcaligenes faecalis	JUO51	1.89	Orange S3
Bacillus cereus	JUO9	2.10	Orange S2
Bacillus cereus	JUO24	2.08	Orange S3
Morganella morganii	JUO60	2.40	Orange S2
Staphylococcus sciuri	JUO10	2.21	Orange S2
Staphylococcus epidermidis	JUO25	2.12	Orange S3
Alcaligenes faecalis	JUP11	2.12	Papaya S1
Alcaligenes faecalis	JUP15	1.84	Papaya S2
Alcaligenes faecalis	JUP64	2.37	Papaya S2
Alcaligenes faecalis	JUP26	2.13	Papaya S3
Bacillus cereus	JUP12	2.19	Papaya S1
Bacillus cereus	JUP54	2.29	Papaya S2
Bacillus cereus	JUP14	1.79	Papaya S2
Bacillus cereus	JUP27	1.81	Papaya S3
Bacillus cereus	JUP28	2.13	Papaya S3
Morganella morganii	JUP52	2.31	Papaya S2
Pseudomonas putida	JUP41	2.02	Papaya S1
Pseudomonas putida	JUP42	1.82	Papaya S2

Where: S1 – Mature green, S2 – Moderately ripe, S3 – Overripe stage,

To establish if the MALDI Biotyper Compass Software could differentiate closely related strains at the genus/species levels, spectra of some recognized isolates were examined and illustrated below (Figure 2).

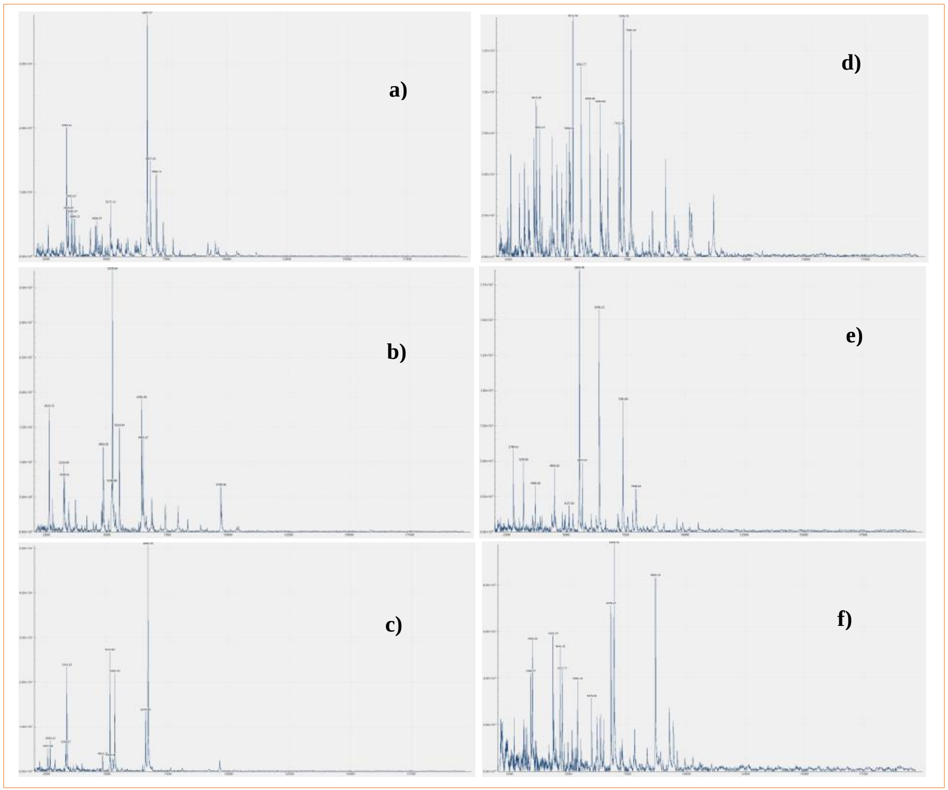

Figure 2. Mass spectrum of Gram-positive and Gram-negative bacterial species: **a)** *B. cereus* in banana-JUB33 (score value= 2.30), **b)** *S. sciuri* in orange- JUO10 (score value= 2.21) and **c)** *S. epidermidis* in orange- JUO25 (score value= 2. 12), and Gram-negative bacterial species: **d)** *A. faecalis* in papaya-JUP64 (score value = 2.37), **e)** *P. putida* in banana-JUB34 (score value = 2.21) and **f)** *M. morganii* in orange- JUO60 (score value = 2.40).

Distribution of dominant bacterial isolates in fruit samples

This study revealed that the bacterial microbiome of all fruit types at different ripening stages was majorly predominated by soil and environmental bacteria. Generally, a total of 243 bacterial isolates grouped into 9 genera were identified from 108 fruit samples collected for this study. The bacterial species identified using the morphological and biochemical tests, as well as the MALD-TOF MS analysis include *B. cereus* (33.7%), *A. faecalis* (17.3%), *P. putida* (15.2%), *M. morganii* (11.1%) and *S. sciuri* (6.6%), found in all fruit samples with different proportions. However, some bacterial species were exceptionally not detected in some fruits at

certain maturity stage. For instance, the opportunistic pathogen *S. epidermidis,* and *E. coli* was not detected on all fruits at mature green stage. Similarly, *Salmonella spp*, *Shigella* spp and *Serratia* spp were not encountered in banana and papaya at moderately ripe stage, and in orange at both mature green and moderately ripe stages. The *Serratia* spp was not detected in all maturity stages of orange, and in banana and papaya at mature green (Table 3). From this result, it could be concluded that the microbial load and diversity in all fruit samples was increasing with ripening; as the lowest microbial load and diversity was observed at mature green stage, and the highest at overripe stage of the fruits.

Table 3. Distribution of dominant bacterial species colonizing fruits at different ripening stages, Bench-Sheko Zone, 2021

Fruit Sample	# Sample	*B. cereus*	*A. faecalis*	*P. putida*	*M. morganii*	*S. sciuri*	*S. epidermidis*	*E. coli*	*Salmonella Spp*	*Shigella Spp*	*Serratia Spp*	Total (%)
Banana S1	12	8 (9.8)	2 (4.8)	2 (5.4)	3 (11.1)	1 (6.3)	-	-	1 (14.3)	1 (16.7)	-	18 (7.4)
Banana S2	12	11 (13.4)	4 (9.5)	3 (8.1)	3 (11.1)	2 (12.5)	3 (25)	1 (10)	-	-	1 (25)	27 (11.1)
Banana S3	12	15 (18.3)	5 (11.9)	5 (13.5)	4 (14.8)	2 (12.5)	2 (16.7)	2 (20)	2 (28.6)	1 (16.7)	1 (25)	39 (16.1)
Orange S1	12	4 (4.9)	4 (9.5)	4 (10.8)	2 (7.4)	1 (6.3)	-	1 (10)	-	-	-	16 (6.6)
Orange S2	12	6 (7.3)	6 (14.3)	5 (13.5)	2 (7.4)	1 (6.3)	2 (16.7)	-	-	-	-	22 (9.1)
Orange S3	12	9 (11)	8 (19)	8 (21.6)	3 (11.1)	2 (12.5)	3 (25)	2 (20)	1 (14.3)	1 (16.7)	-	37 (15.2)
Papaya S1	12	7 (8.5)	3 (7.1)	3 (8.1)	3 (11.1)	2 (12.5)	-	1 (10)	1 (14.3)	1 (16.7)	1 (25)	22 (9.1)
Papaya S2	12	10 (12.2)	4 (9.5)	3 (8.1)	3 (11.1)	3 (18.75)	1 (8.3)	-	-	-	-	24 (9.9)
Papaya S3	12	12 (14.6)	6 (14.3)	4 (10.8)	4 (14.8)	2 (12.5)	2 (16.7)	3 (30)	2 (28.6)	2 (33.3)	1 (25)	38 (15.6)
Total	108	82 (33.7)	42 (17.3)	37 (15.2)	27 (11.1)	16 (6.6)	12 (4.9)	10 (4.1)	7 (2.9)	6 (2.4)	4 (1.6)	243 (100)

Where: S1 – Mature green, S2 – Moderately ripe, S3 – Overripe stage, Number of isolates in columns – Frequency (%)

Distribution of dominant fungal isolates in fruits samples at different ripening stages

In this study, a total of 375 fungal isolates (molds and yeasts) grouped into 9 genera were isolated from 108 fruit samples and identified using morphological and biochemical tests. The results of this study shown that the fungal microbiome of the fruit samples at different ripening stages was predominated by *Candida* spp (33.9 %), *Saccharomyces* spp (18.1 %), *Aspergillus* spp (16.3 %), *Alternaria* spp (8.5), *Penicillium* spp (7.2%) and *Fusarium* spp (6.7%); which were detected in all fruit samples with different proportions (Table 3). Still, the frequencies of isolation of other fungi differs in different maturity stages of the fruits. More specifically, *Alternaria* spp (18.7%) were dominant in mature green papaya, while *Penicillium* spp (22.2%) in overripe orange and *Fusarium* spp (24%) dominated the overripe banana. Overall, the least number of *Mucor* spp (3.2) and *Rhizopus* spp (2.7) were observed in the tested fruit samples. Whereas, *Fusarium* spp were not detected in mature green orange, as the case is for *Botrytis* spp in mature ripe papaya, and moderately ripe and overripe banana (Table 4). This result indicates that similar to the bacterial community, the fungal load and diversity in all fruit samples was increasing with ripening; as the lowest fungal load and diversity was observed at mature green stage, and the highest at overripe stage of the fruits.

Table 4. Distribution of dominant mold and yeast isolates in fruit samples at different ripening stages, Bench-Sheko Zone, 2021

Sample Type	Sample size	*Candida* spp.	*Saccharomyces* spp	*Aspergillus* spp.	*Alternaria* spp.	*Penicillium* spp.	*Fusarium* spp.	*Botrytis* spp.	*Mucor* spp.	*Rhizopus* spp.	Total (%)
Banana S1	12	13 (10.2)	8 (11.8)	6 (9.8)	3 (9.4)	2 (7.4)	3 (12)	2 (15.4)	4 (33.3)	2 (20)	43 (11.5)
Banana S2	12	14 (11)	8 (11.8)	8 (13.1)	2 (6.3)	1 (3.7)	4 (16)	-	-	-	37 (9.9)
Banana S3	12	16 (12.6)	9 (13.2)	9 (14.8)	5 (15.6)	3 (11.1)	6 (24)	-	2 (16.7)	2 (20)	52 (13.9)
Orange S1	12	12 (9.5)	5 (7.4)	4 (6.6)	2 (6.3)	2 (7.4)	-	2 (15.4)	-	1 (10)	28 (7.5)
Orange S2	12	13 (10.2)	6 (8.8)	5 (8.2)	4 (12.5)	4 (14.8)	1 (4)	2 (15.4)	-	-	35 (9.3)
Orange S3	12	13 (10.2)	7 (10.3)	7 (11.5)	3 (9.4)	6 (22.2)	3 (12)	3 (23.1)	1 (8.3)	1 (10)	44 (11.7)
Papaya S1	12	13 (10.2)	7 (10.3)	6 (9.8)	6 (18.7)	1 (3.7)	1 (4)	3 (23.1)	1 (8.3)	-	38 (10.1)
Papaya S2	12	16 (12.6)	9 (13.2)	8 (13.1)	3 (9.4)	3 (11.1)	3 (12)	-	-	2 (20)	44 (11.7)
Papaya S3	12	17 (13.4)	9 (13.2)	8 (13.1)	4 (12.5)	5 (18.5)	4 (16)	1 (7.7)	4 (33.3)	2 (20)	54 (14.4)
Total	108	127 (33.9)	68 (18.1)	61 (16.3)	32 (8.5)	27 (7.2)	25 (6.7)	13 (3.5)	12 (3.2)	10 (2.7)	375 (100)

Where, S1 – Mature green, S2 – Moderately ripe, S3 – Overripe stage, Number of isolates in columns – Frequency (%)

Commented [Rev14]: What is in parenthesis denotes

Physico-chemical properties of fruit samples at different ripening stages

Peel color

In accordance with the standard scale [25], that categorizes the maturity level of banana, only three of the seven stages: stage 1 (mature green), stage 5 (yellow with green tips), and stage7 (yellow with brown spots) were considered in this study. For orange and papaya fruit samples, mature green, moderately ripe (yellow with a trace of green), and overripe (fully yellow) were considered. Peel color of the samples was matched to a standard reference key and documented by photographing (Figure 3).

Mature Green Moderately ripe Overripe

Banana

Orange

Papaya

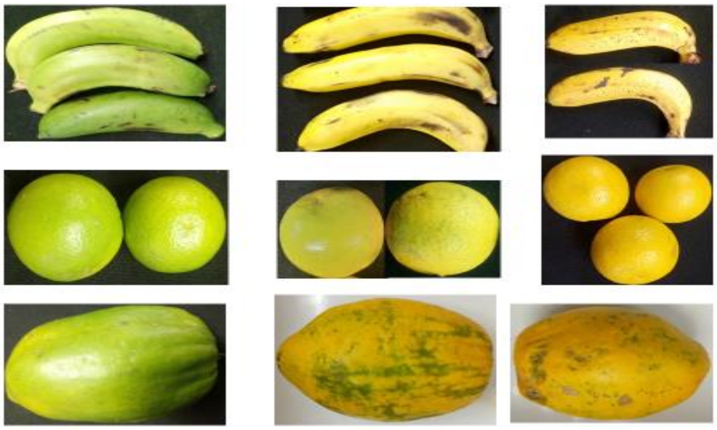

Figure 3: Peel color and maturity level of the fruits samples (banana, orange and papaya)

Physico-chemical composition of fruit samples

The present study revealed that as ripening stage increased, the fruits showed significantly decreased pH value and increased TA and moisture content. Among the fruit samples, relatively orange was more acidic (pH, 3.53 - 4.03) than banana (pH, 4.47 - 5.73) and papaya (5.72 - 6.08). Moreover, the highest TA (1.28 mg/100g) was recorded in mature green oranges.

Table 5. Physico-chemical properties (mean ± S.D.) of fruits samples at different ripening stages, Bench-Sheko Zone, 2021

	**Banana *(Musa paradisiaca)***			**Orange *(Citrus sinensis)***			**Papaya *(Carica papaya)***		
**Tests**	**S1**	**S2**	**S3**	**S1**	**S2**	**S3**	**S1**	**S2**	**S3**
pH	5.73 ± 0.15^{a}	4.70 ± 0.10^{b}	4.47± 0.06^{c}	3.53 ± 0.06^{b}	3.63 ± 0.06^{b}	4.03 ±0.21^{a}	6.08 ±0.07^{a}	5.72 ± 0.07^{b}	5.67 ± 0.04^{b}
TA (mg/100g)	0.46 ± 0.04^{a}	0.91 ± 0.02^{b}	1.03 ± 0.08^{c}	1.28 ± 0.04^{a}	0.92 ± 0.02^{b}	0.75 ±0.03^{c}	0.03 ± 0.01^{c}	0.15 ± 0.01^{a}	0.10 ± 0.01^{b}
Moisture (%)	71.40 ± 0.95^{a}	74.33 ± 0.40^{b}	75.77 ± 0.38^{c}	86.20 ± 0.60^{b}	88.73 ± 0.35^{a}	85.53 ±0.83^{b}	87.27 ± 0.97^{b}	90.73 ± 1.60^{a}	92.37 ± 0.95^{a}
SC (%)	28.60 ± 0.95^{a}	25.67 ± 0.40^{b}	24.03 ± 0.72^{c}	13.80 ± 0.60^{a}	11.27 ± 0.35^{b}	14.47 ±0.83^{a}	12.73 ± 0.97^{a}	9.23 ± 1.60^{b}	7.63 ± 1.14^{b}
AA (mg/100g)	6.32 ± 0.08^{b}	8.10 ± 0.22^{a}	6.40 ± 0.05^{b}	39.77 ± 0.91^{b}	49.50 ± 0.70^{a}	40.77 ±1.39^{b}	61.63 ± 0.85^{c}	68.13 ± 0.31^{b}	69.87 ± 0.31^{a}
RS (%)	0.00 ± 0.00^{c}	12.13 ± 0.40^{b}	14.20 ± 0.40^{a}	4.07 ± 0.07^{c}	5.90 ± 0.26^{b}	8.87 ±0.31^{a}	4.63 ± 58^{b}	6.07 ± 0.03^{a}	6.30 ± 0.05^{a}
TS (%)	0.01 ± 0.01^{c}	15.20 ± 0.40^{b}	17.87 ± 0.31^{a}	6.80 ± 0.40^{c}	10.65 ± 0.79^{b}	12.73 ±0.61^{a}	5.22 ± 0.45^{c}	7.01 ± 0.20^{b}	7.88 ± 0.02^{a}

Commented [Rev15]: Provide CV value and F value in table

AA = Ascorbic acid, TA = Titratable acidity, SC= Solid content, AA = Ascorbic Acid, RS = Reducing sugar, TS = Total Sugar. Different letters along figures indicate a significant difference ($p < 0.05$); the same letters indicate no significant difference ($p > 0.05$).

On the other hand, the moisture content (%) increased as the ripening stage progressed from mature green (71.40 ± 0.95-87.27 ± 0.97) to overripe (75.77 ± 0.38-92.37 ± 0.95) in all fruits. The highest content of ascorbic acid (mg/100g) was observed in papaya (61.63 - 69.87) followed by orange (39.77 - 49.50) and banana (6.32 8 - 8.10). Also, significant increment in the amounts of total sugar (0.01 ± 0.01-17.87 ± 0.31) and reducing sugar (0.00 ± 0.00-14.20 ± 0.40) were recorded in banana and doubling in the amount of total sugar (6.80 ± 0.40-12.73±0.61) in orange with progress in the ripening stages (Table 5).

Discussions

The microbial enumeration data in the present study showed that there was a continuous increment in the microbial load as ripening progresses in the fruit samples. The mean minimum and maximum counts (Log $CFUg^{-1}$) of AMB were noted at the mature green and overripe stages of all fruit samples. Accordingly, mean count (Log $CFUg^{-1}$) recorded from banana (6.76-7.96), and orange (6.74-7.51) were in consistence with previous reports made by Fajinmi & Aduramigba-Modupe (2011) and Aneja et al., (2014), respectively. The increasing microbial load in fruits in due course of maturation is probably due to the continuous increment of nutritional content of the fruits and time of exposure to vectors associated with fruits [35].

In the present study, 36 bacterial isolates from top-five most dominant strains were randomly selected as representative, among which 97.2% of them were identified to species/genera level using MALDI-TOF MS. Accordingly, the bacterial community of all fruit samples at different ripening stages was observed dominated by *B. cereus* (33.7%), *A. faecalis* (17.3%), *P. putida* (15.2%), *M. morganii* (11.1%), *S. sciuri* (6.6%) and *S. epidermidis* (4.9%). Majority of the identified isolates in this study were soil and environmental bacteria. In agreement with our observation, Abdullah et al., (2017) *also* reported that *Bacillus* spp, *Staphylococcus* spp, and *E. coli* were dominant in fruits. The highest number of *Bacillus* spp, and *Staphylococcus* spp recorded in the study could be due to versatile metabolism, cross-contamination with soil carried by insects and birds vectors, and humans during farming [36]. The presence of enteric microorganisms in fruits could be due to the addition of human fecal matter and animal manure in the traditional farm sites, and bird and insect vector interaction (use fruit plants as a nest) [35].

Moreover, the fungal microbiome of fruit samples at all ripening stages was dominated by *Candida* spp (33.9%), followed by *Saccharomyces* spp (18.1%) and *Aspergillus* spp (16.3%). Likewise, fungal microbiome of orange samples was reported to constitute *Candida* spp [34], *Saccharomyces* spp [37], *Aspergillus* spp. and *Penicillium,* spp. [38]. Furthermore, different ripening stages of banana and papaya fruits were observed to be dominated by *Candida* spp., *Saccharomyces* spp., *Aspergillus* spp., and *Alternaria* spp. [39, 40]. This result shows that the fruit at harvest stages were contaminated by key fungal postharvest pathogens which could result in spoilage of fruits [12]. Fruits can harbor relatively high numbers of microorganisms at harvest as normal microflora because of their potential contact with water and soil during growth [36].

In this study, the pH values the fruits were significantly decreased with progresses in maturity as observed in banana (5.73 to 4.47) and papaya (6.08 to 5.67) while TA values increased significantly as clearly observed in banana (0.46%, at mature green, to 1.03% at overripe stage). The finding is in line with the report of Kumalasari et al., (2021). The same increment patterns were observed for papaya (from 0.03% to 0.15% at mature green and overripe stage, respectively), results supported by previous reports [24, 42]. In contrary, significant decline ($p<0.05$) in TA values with progress in maturity stages (1.28% to 0.75 %, at mature green and overripe stage, respectively) were observed in orange samples. This was in line with the findings of Riaz et al., (2015). As a result, the pH value of the orange samples has significantly increased ($p<0.05$), with values ranging between 3.53 (mature green stage) to 4.03 (overripe stage). This finding is consistent with the findings of Gloria et al, (2010). In citrus fruits, the cause for the decrease in acidity during the ripening might be due to the utilization of citric acid in the fruit respiratory process [44]. Fruits at green stage mainly contain starch, though the degradation of starch with progress in ripening may result in increase in sugars along with malic acid, citric acid and oxalic acid. This, in turn, results in an increase in TA content and decrease in pH values [45].

The mean moisture content in the analyzed banana fruits showed a significant increase ($p<0.05$) with ripening stage (71.40%, at mature green, to 75.77% at overripe stage). This result is similar to the findings reported by Phillips et al (2021). In papaya samples, too, it increased from 87.27% (at mature green) to 92.37% at overripe stage. This finding is in line with the result reported in Taiwan [40] and Malaysian papaya [48].The high tendency of retaining and rise in moisture contents of these fruits is due to their ability to keep moisture content longer than other

plants [48]. On the other hand, the moisture content of orange fruit samples showed a significant increase ($p<0.05$) as ripening progressed from mature green (86.20%) to moderately ripe (88.73%) stage, but decreased at overripe stage (85.53%). This result is consistent with the findings of Gloria et al (2010) who conducted the analysis on Valencia oranges. The decrease in moisture content after mid stage of ripening is due to the onset of new fruit growth and development which suck back the moisture and nutrients from the old fruits of orange plant [26].

Ascorbic acid content in banana samples was significantly increased ($p<0.05$) with progress in maturity from (6.32 mg/100g) mature green to moderately ripe stage (8.10 mg/100g) but decreased later at overripe stage (6.40 mg/100g). Similar results were also reported by Borges et al. (2019). Likewise, the ascorbic acid content of orange increased with progress from mature green (39.77 mg/100g) to moderately ripe stage (49.50 mg/100g) though decreased significantly to 40.77 mg/100g at overripe stage. The same pattern in content of ascorbic was reported by Gloria et al, (2010) in a study conducted on Valencia orange. The decrease in ascorbic acid content after mid-stage of ripening is due to enzymatic reactions and formation of dehydro-ascorbic acid from the oxidation of ascorbic acid, during over ripening [27]. In contrast the above two fruits, the Bench-Sheko papaya fruits presented continuous and significant changes ($p<0.05$) in ascorbic acid content during ripening stage from mature green (61.63 mg/100g) through moderate ripe (68.13 mg/100g) to overripe stage (69.87 mg/100g). This finding is consistent with the result reported by Ovando-Martinez et al., (2018) indicating the ascorbic acid content would significantly increase with progress in maturity of papaya.

The Bench-Sheko banana samples showed a significant increase ($p<0.05$) in both reducing and total sugar contents, as ripening progressed. The reducing sugar and total sugar contents were, respectively, 0.00% and 0.01% (mature green), 12.13% and 15.2% (moderately ripe) and 14.20% and 17.87% (overripe stage). Other studies also reported related dynamics in sugar contents with advance in ripening stages of banana [36, 46]. Similarly, mean reducing sugar content in orange fruit ranged from 4.07% (mature green), through 5.90% (moderately ripe) to 8.87% (overripe stage), values in agreement observation and report made by Gloria et al, (2010). The total sugar contents in orange were also significantly higher both during moderately ripe (10.65%) and overripe (12.73%) maturity stages as compared to the mature green stage (6.80 %), consistent with the finding of Gloria et al, (2010). Likewise, the mean reducing sugar contents of papaya

was increased from 4.63% at mature green, to 6.07% at moderately ripe and 6.30% at overripe stage, results in agreement with report made from papaya analyzed in Indonesian [51]. Moreover, the total sugar contents of papaya samples increased from 5.22% (mature green), through 7.01% (moderately ripe) to 7.88% (overripe stage). This result is strongly supported by the findings of Kamelia et al (2019). The increment in soluble sugars results from starch degradation after seed maturation which is accompanied by increased activity of sucrose synthase during fruit ripening [52].

Conclusions

Commented [Rev16]: Conclusion should be more focused and further recommendations

With progress in the ripening stages of all fruits analyzed in this study, the microbial counts, diversity of microbiomes, Physico-chemical parameters, ascorbic acid, and total carbohydrate contents changed significantly. Accordingly, the microbial counts were significantly increased with ripening stage in all fruits, except counts of *Enterobacteriaceae* in banana and orange and fungal counts in orange fruit samples. Most importantly, this study revealed the association of diverse microbiome including human and fruit pathogens such as *B. cereus, A. faecalis, M. morganii, S. epidermidis*, *Candida* spp, *Aspergillus* spp, *Alternaria* spp, *Penicillium* spp, and *Fusarium* spp in Bench-Sheko fruit samples. Bench-Sheko zone is well known for its production and distribution of fruits to different areas of the country. Hence, considering an appropriate management system starting from farming to collection, distribution, and consumption of the fruits is critical important to ensure safety of the consumers and minimize product losses.

Acknowledgments

The authors would like to thank Jimma University, College of Natural Sciences for funding; Jimma University, Department of Biology, Ethiopian Ministry of Education, and Mizan–Teppi University for facilitating the study to DK.

Authors' contributions

DK: Conceptualization, designing, validation, formal analysis, investigation, resources, data curation, writing the original draft. **KB:** Conceptualization, designing, validation, formal analysis, reviewing and editing, supervision, project administration, and fund acquisition. **LL:**

Conceptualization, designing, validation, formal analysis, resources, investigation. **RN**: Conceptualization, designing, validation, formal analysis, investigation, reviewing and editing original draft. The authors read and approved the final manuscript.

Funding

The work was undertaken as part of mega project funded by Jimma University under grant agreement No. 81794 (to KB).

Availability of data and materials

All data generated during this study are included in this article.

Declarations

Ethics approval and consent to participate

Not applicable.

Consent for publication

Not applicable.

Competing interests

The authors declare that they have no competing interests.

Author details

[1]Jimma University, College of Natural Sciences, Department of Biology, Jimma, P.O. Box 378, Ethiopia,

[2]Mizan-Teppi University, College of Natural and Computational Sciences, Department of Biology, Teppi, P.O. Box 126, Ethiopia;

[3]Dembi Dollo University, College of Natural and Computational Sciences, Department of Biology, Dembi Dollo, P.O. Box 260, Ethiopia.

6. References

1. Batista-Silva W, Nascimento VL, Medeiros DB, Nunes-Nesi A, Ribeiro DM, Zsögön A, et al. Modifications in organic acid profiles during fruit development and ripening: Correlation or causation? Front Plant Sci. 2018;871:1–20.
2. FAO. Fruit and vegetables – your dietary essentials. The International Year of Fruits and Vegetables, 2021, background paper. Rome.; 2020.
3. FAO. Global food losses and food waste – Extent, causes and prevention. 2011;11(11):38. Available from: http://www.fao.org/fileadmin/user_upload/ags/publications/GFL_web.pdf
4. USDA GAIN Report. Ethiopia fresh fruits market update report. FAS Addis Ababa. 2018;1827:1–8.
5. IDRC. Canada's International Development Research Centre - CIFSRF final technical report: Enhanced Preservation of Fruits Using Nanotechnology (CIFSRF Phase 2). 2018;1–90. Available from: https://idl-bnc-idrc.dspacedirect.org/handle/10625/57175
6. Rawat S. Food Spoilage: Microorganisms and their prevention. Pelagia Res Libr Asian J Plant Sci Res (Internet). 2015;5(4):47–56. Available from: www.pelagiaresearchlibrary.com
7. Seymour GB, Ostergaard L, Chapman NH, Knapp S, Martin C. Fruit development and ripening. Annu Rev Plant Biol. 2013;64:219–41.
8. Gao J, Zhang Y, Li Z, Liu M. Role of ethylene response factors (ERFs) in fruit ripening. Food Qual Saf. 2020;4:15–20.
9. Berendsen RL, Pieterse CMJ, Bakker PAHM. The rhizosphere microbiome and plant health. Trends Plant Sci. 2012;17:478–86.
10. Poudel M, Mendes R, Costa LAS, Bueno CG, Meng Y, Folimonova SY, et al. The Role of Plant-Associated Bacteria, Fungi, and Viruses in Drought Stress Mitigation. Front Microbiol. 2021;12(October):1–21.
11. Buchholz F, Kostić T, Sessitsch A, Mitter B. The potential of plant microbiota in reducing postharvest food loss. Microb Biotechnol. 2018;11(6):971–5.
12. Zhang W, Li Z, Du M, Zhang X, Tian Y, Wang J. 1-Methylcyclopropene (1-MCP) retards the senescence of Pteridium aquilinum var. latiusculum by regulating the cellular energy status and membrane lipid metabolism. Food Sci Nutr. 2021;9:4349–63.
13. Wedajo B, Kadire A. Assessment of Bacterial Load of Some Fresh and Packed Fruit Juices in Arba Minch Town, Ethiopia. J Nutr Food Sci. 2019;09(03):1–7.
14. Weldezgina D, Muleta D. Bacteriological Contaminants of Some Fresh Vegetables Irrigated with Awetu River in Jimma Town, Southwestern Ethiopia. Adv Biol. 2016;2016:1–11.
15. CSA. the Federal Democratic Republic of Ethiopia Central Statistical Agency Report on Area and Production of Crops. Fed Democr Repub Ethiop Cent Stat Agency. 2021;V. I(April 2021):128.
16. Tesfaye AA and B. Review on Assessment of Horticultural Crops Production, Constraints

and Opportunities in Ethiopia. Int J Agric Biosci. 2019;8:89–98.

17. Bekele G, Kebede K, Ameha N. Study of Indigenous Chicken Production System in Bench Maji Zone, South Western Ethiopia. Glob J Sci Front Res D Agric Vet. 2016;16.

18. Kompaníková J, Neuschlová M, Sadloňová V. Univerzita Komenskeho V Bratislave Jesseniova Lekarska Fakulta V Martine Special Bacteriology Basic Laboratory Tests. Univ Comeniana Bratisl Fac Medica Jesseniana. 2016;6–13.

19. Al-mohanna MT. METHODS FOR FUNGAL ENUMERATION , ISOLATION AND C H A P T E R 7. In: ORIGIN OF OTA. 2017. p. 155–241.

20. Cheesbrough M. District Tropical Practice in Laboratory Countries. Second Edi. Cheesbrough M, editor. CAMBRIDGE UNIVERSITY PRESS; 2006.

21. Zhang R, Zhang Y, Zhang T, Xu M, Wang H, Zhang S, et al. Establishing a MALDI-TOF-TOF-MS method for rapid identification of three common Gram-positive bacteria (Bacillus cereus, Listeria monocytogenes, and Micrococcus luteus) associated with foodborne diseases. Food Sci Technol. 2022;42:1–9.

22. Ellis D, Davis S, Alexiou H, Handke R BR. Descriptions of Medical Fungi. Vol. 9, Australasian Medical Journal (Online). 2016. 296 p.

23. Zafar A, Jabeen K, Farooqi J. Practical guide and atlas for the diagnosis of fungal infections. The Aga Khan University; 2017. 1–68 p.

24. Soto M, Brenes M, Jiménez N, Cortés C, Umaña G, Pérez AM. Selection of optimal ripening stage of papaya fruit (Carica papaya L.) and vacuum frying conditions for chips making. CYTA - J Food. 2021;19:273–86.

25. Mazen FMA, Nashat AA. Ripeness Classification of Bananas Using an Artificial Neural Network. Arab J Sci Eng. 2019;44:6901–10.

26. Riaz M, Zamir T, Rashid N, Jamil N, Rizwan S, Masood Z, et al. Comparative Study of Nutritional Quality of Orange (Citrus sinensis) at Different Maturity Stages in Relation to Significance for Human Health. Am-Euras J Toxicol Sci. 2015;7:209–13.

27. Borges CV, Amorim EP, Leonel M, Gomez HAG, Santos TPR dos, Ledo CA da S, et al. Post-harvest physicochemical profile and bioactive compounds of 19 bananas and plantains genotypes. Bragantia. 2019;78(2):284–96.

28. AOAC. Official Method 934.06. Codex-Adopted–AOAC Method. Fruits. 2000;215(March 1996):2000–2000.

29. AOAC. AOAC Official Method 942.15. AOAC Official Methods of Analysis. 2000.

30. Matabura VV, Kibazohi O. Physicochemical and Sensory Evaluation of Mixed Juices from Banana, Pineapple and Passion Fruits during Storage. Tanzania J Sci (Internet). 2021;47(1):332–43. Available from: https://dx.doi.org/10.4314/tjs.v47i1.28

31. AOAC. AOAC Official Method 923.09. Lane-Eynon General Volumetric Method: Invert Sugar in Sugars and Syrups. 2000;35(1923):8013–8013.

32. FSSAI. Manual of Methods of Analysis of Foods Fruit and Vegetable Products Food Safety and Standards Authority of India Manual for Analysis of Fruit and Vegetable Products. Food Saf Stand Auth India. 2016;01:1–86.

33. Fajinmi O, Akinyemi S. Influence of storage methods on ripening and microbial load of some Musa species. Agric Biol J North Am. 2011;2(8):1201–7.

34. Aneja KR, Dhiman R, Aggarwal NK, Kumar V, Kaur M. Microbes associated with freshly prepared juices of citrus and carrots. Int J Food Sci. 2014;2014:1–7.

35. Abdullah R, Qaiser H, Farooq A, Kaleem A, Iqtedar M, Aftab M, et al. Avaliação do potencial microbiana e amadurecem comportamento de bananas preclimacteric seguinte irradiação gama. Biosci J. 2017;33:57–65.

36. Jahan N, Noor R, Munshi SK. Microbiological analysis and determination of antimicrobial traits of green banana (Musa spp.) and papaya (Carica papaya). Stamford J Microbiol. 2019;8:41–5.

37. Obasi, Whong A and E. Microbiological Quality Assessment of Commercially and Laboratory Prepared Orange Juice. J Biotechnol Res. 2019;5:19–27.

38. Muhammad, IU M, M A, I B, BS H, HA B, et al. Isolation and identification of fungi associated with the spoilage of sweet orange (Citrus sinensis L) and banana (Musa sapientum L) in Sokoto Metropolis. J Appl Biotechnol Bioeng. 2018;5(3):172–82.

39. Krishnan P, Bhat R, Kush A, Ravikumar P. Isolation and functional characterization of bacterial endophytes from Carica papaya fruits. J Appl Microbiol. 2012;113:308–17.

40. Kher RM, Sahu FM. Engineering physical properties of papaya (Carica papaya L) at different ripening stages. Int J Chem Stud. 2018;6:1945–52.

41. Kumalasari R, Vanadiani L, Ekafitri R, Nurminabari IS, Desnilasari D, Indah Mayasti NK, et al. Properties of Indonesian plantain cultivars during ripening and recommendation for flour ingredients. Czech J Food Sci. 2021;39:35–41.

42. Addai ZR, Abdullah A, Mutalib SA, Musa KH, Douqan EMA. Antioxidant activity and physicochemical properties of mature papaya fruit (Carica papaya L. cv. Eksotika). Adv J Food Sci Technol. 2013;5(7):859–65.

44. Gloria P, Mubofu Egid B, Othman Chande C. Post harvest changes in physico-chemical properties and levels of some inorganic elements in off vine ripened orange (Citrus sinensis) fruits cv (Navel and Valencia) of Tanzania. African J Biotechnol. 2010;9:1809–15.

45. Ahalya C, Ip S, Ganapathy S, Aruna P, Geethalakshmi I. Studies on physico-chemical changes in hill bananas during ripening. J Pharmacogn Phytochem. 2021;10:1791–5.

46. Phillips KM, McGinty RC, Couture G, Pehrsson PR, McKillop K, Fukagawa NK. Dietary fiber, starch, and sugars in bananas at different stages of ripeness in the retail market. PLoS One (Internet). 2021;16(7 July):1–21. Available from: http://dx.doi.org/10.1371/journal.pone.0253366

48. Zuhair RA, Aminah A, Sahilah AM, Eqbal D. Antioxidant activity and physicochemical properties changes of papaya (Carica papaya L. cv. Hongkong) during different ripening stage. Int Food Res J. 2013;20:1653–9.

49. Ovando-Martinez M, López-Teros V. M, Tortoledo-Ortiz O, Astiazarán-García H, Ayala-Zavala JF, Villegas-Ochoa MA, et al. Effect of ripening on physico-chemical properties

and bioactive compounds in papaya pulp, skin and seeds. Indian J Nat Prod Resour. 2018;9(1):47–59.

51. Kamelia M, Supriyadi, Widiani N, Farida, Damayanti I. The Analysis of Glucose and Vitamin C on Papaya (Carica papaya L.) with Different Time Storage. J Phys Conf Ser. 2019;1338(1).

52. Zhou L, Paull RE. Sucrose metabolism during papaya (Carica papaya) fruit growth and ripening. J Am Soc Hortic Sci. 2001;126:351–7.
