# Supplementary material for: Evaluation of microbiome and physico-chemical profiles of fresh fruits of Musa paradisiaca, Citrus sinensis and Carica papaya at different ripening stages: Implication to quality and safety management
Source: PLoS One. 2024 Jan 30;19(1):e0297574. doi: 10.1371/journal.pone.0297574 (PMC10826968; doi:10.1371/journal.pone.0297574)
Supplement: Attachment — Submitted filename: Response to Reviewers.docx [file pone.0297574.s009.docx]

Dear Dr. Pandey, Academic Editor of PLOS ONE Journal and Reviewer, PLOS ONE Journal,

First of all, we want to reflect our heartfelt gratitude and appreciation for all the comments and questions you provided while reviewing our manuscript. We found them highly constructive and basic. Thus, we have tried to carefully address all the comments and questions. We have incorporated the comments and enquires; and the corrections and modifications are indicated as marked yellow in the revised version of the manuscript. Except some editorial corrections, all of them are briefed below.

Best regards!

The authors.

**Comments and enquires from the Academic Editor,**

1. We have carefully reviewed the PLOS ONE style templates, and made modifications in our manuscript to confirm that it meets PLOS ONE's style requirements.

2. Regarding the enquires to provide information regarding the permits we obtained for the work, a research approval and support letter from Jimma University, written to all concerned bodies was obtained and reported to the local administrations of the study sites. All concerned bodies have permitted access to the work site. However, no written approval letters were required since the study was not related to medical, veterinary or socio-demographic aspects of the residents. Detailed information is included as “Ethical permission issues” in the revised manuscript.

3. Concerning our grant information provided in the ‘Funding Information’ and ‘Financial Disclosure’ sections, we have reconsidered and made modifications accordingly.

4. About our Data Availability statement, the comments were fully accepted and the related data are uploaded as “Supporting Information” files with the revised version of the manuscript.

5. Regarding the map of the study site (Figure 1) in our submission, the map is our original work, and it is not downloaded from any web site. We constructed the map using the ArcGIS 10.8 software. As indicated in the revised manuscript, the shapefiles used to construct the map could be downloaded from <https://data.humdata.org/dataset/cod-ab-eth>.

However, according to your recommendation, we have tried to communicate the case with the USGS National Map Viewer (the only responding website). So far, they replied that they provide a permission statement only if the map product was downloaded from their page.

Finally, as recommended, all figures in our revised manuscript are converted to the .tiff mode following the Preflight Analysis and Conversion Engine (PACE) digital diagnostic tool.

**Comments and enquires from the Reviewer,**

**[Rev4 (Page 3)]:** Indicates the introduction part needs to strengthen. We accepted and revised it considering all the recommendation as marked in the text.

**[Rev5 (Page 4)]:** Mentions to rewrite clear objectives. We have accepted and incorporated accordingly, marked yellow in the text.

**[Rev6 (Page 5)]:** Comments to show the surveyed places in the Map. The three surveyed sites are zoomed out and shown separately in the map.

**[Rev7 (Page 6)]:** Questions to include author name for each fruit. The comment is considered and indicated in the text.

**[Rev8 (Page 7)]:** Recommends to write details about characterization, and to reason out why we didn’t do molecular characterization of the microbes. Unfortunately, we couldn’t conduct the molecular identification of the microbes due to lack of access to molecular lab facilities in the country; and shortage of budget was another constraint even to send the isolates abroad for the analysis. Therefore, we used a relatively new MALD – TOFF MS technology to identify the isolates at genus/species level.

**[Rev9 (Page 8)]:** Enquires if the experiments repeated or performed in replications. All the experiments performed in replications, and indicated in the text.

**[Rev11 (Page13)]:** Questions How did we handle data from repeated experiments, if we do homogeneity and normality test, to clarify what kind of data we used, raw data or transformed data in analysis. The questions are carefully considered and answers are included accordingly.

**[Rev12 (Page13)]:** Comments that all the results must be supported with statistical value. The comment is accepted and incorporated accordingly.

**[Rev15 (Page23)]:** Enquires to provide CV value and F value in table (Table 5). Well accepted and incorporated in all tables with similar correction.
