# Supplementary material for: Evaluation of microbiome and physico-chemical profiles of fresh fruits of Musa paradisiaca, Citrus sinensis and Carica papaya at different ripening stages: Implication to quality and safety management
Source: PLoS One. 2024 Jan 30;19(1):e0297574. doi: 10.1371/journal.pone.0297574 (PMC10826968; doi:10.1371/journal.pone.0297574)
Supplement: Attachment — Submitted filename: Response to Reviewers.docx [file pone.0297574.s010.docx]

Dear,

Dr. Abhay K. Pandey, Academic Editor, the PLOS ONE Journal

Dr. Nii Korley Kortei, Reviewer, the PLOS ONE Journal and

Dr. Nirmal Kumar Meena, Reviewer, the PLOS ONE Journal,

First of all, we (the authors) want to express our earnest gratitude for all your constructive comments and enquiries you provided while reviewing our manuscript. We believe that all the efforts are made for the betterment of our work before publication. In the present revision, we have tried to carefully address all the comments and questions currently raised by the Academic Editor and the reviewers at the PLOS ONE Journal. The resulting modifications are shown as highlighted yellow in the revised version of the manuscript separately attached labeled as ‘Responses to Reviewers’.

Warm regards!

**Comments and enquires from Reviewers,**

1. Climacteric fruits should be harvested at fully mature stage whereas non climacteric fruits should be harvested after ripening at tree itself.

- That is definitely right, and we did it. All the fruit samples at all ripening stages (including the non-climacteric orange fruit) used in this study were directly collected from farms of village residents, freshly cut from trees. That is why we collected samples by three phases of the needed ripening stages of the fruits (mature green, moderately ripe and overripe). All done aseptically.

2. In case of citrus, how can one determine overripe stage? Please make it clear of ripening stage.

- In order to obtain orange fruit samples at different ripening stages, we used rind coloration level. In case of citrus fruits, specific scores have been developed by using an objective color charts, from 1 (deep reddish orange) to 8 (dark-green) (le Roux 2006). In this study, orange fruits with color score 1 (deep reddish orange) was considered as overripe.

**Reference:** Smit le Roux, 2006. Preharvest manipulation of rind pigments of Citrus spp. Page 187.

3. Other most important fact is that the microbial load over the fruit surface directly associated with the surrounding microclimate, harvest practices, and cultural operations which have been followed. There may be chances of contaminations at the time of harvesting but during handling operations we can minimize the contamination by several postharvest treatments. Why to go up to overripe stage?

- As you have frankly said, the microbial load over the fruit surface directly associated with the surrounding microclimate, harvest practices, and cultural operations. This work is an original work us, since the microbiota and physico-chemical aspects of fresh cut fruits that can be affected by the surrounding microclimate, harvest practices, and cultural operations in the study area wasn’t assessed prior to this study. Additionaly, in this study, it is not only microbial load, but also alterations in microbial diversity and changes in physic-chemical properties of fruits at different ripening stages have been considered. The objective of this study was to evaluate the alterations in microbiome and physico-chemical properties of fresh cut fruits at different ripening levels (mature green, moderately ripe and overripe stages). At country level, we doubt that no study has been conducted at this level on microbiota of fruits.
- Why we have gone up to overripe stage is to track on the alteration in microbiome profiles and physico-chemical properties of fruits at different ripening stages. This has also helped us to track the occurrences of human pathogens and the dominant spoilage microorganism causing fruit decay, and well identified. The application of green nanotechnology to alleviate loss of fruits and health problems occurring due to the presence of these microbes will be the next work of our project.

4. This is obvious that overripe fruits highly prone to microbial attack due to soft skin and less firmness along with poor defense system. The major infecting pathogens in each fruit were already established by several researchers.

- Previously, several studies have been conducted concerning only human and/or plant pathogens focusing only on fruit samples from markets, or partially spoiled fruit samples, still with limited parameters. The study of alterations in microbiome (microbial ecology) and physico-chemical profiles in fruits at different ripening stages was not conducted in depth, especially in Ethiopia.

5. Furthermore, author did not estimate defense related attributes as they play crucial role in delaying decay and infection process. So, please make it clear and establish a clear-cut hypothesis behind the research.

- As a Ph.D. candidate, this manuscript is the first objective of my graduate dissertation. The project is of a wide scope, stretched up to preparation and application of food preservatives using green nanotechnology. Therefore, some *in vitro* and *in vivo* reactions of the identified microbes against the prepared nano-materials and the defense related attributes will be evaluated in the next series of study results under process.
